# Molecular mimicry between tumor associated antigens and microbiota-derived epitopes

**DOI:** 10.1186/s12967-022-03512-6

**Published:** 2022-07-14

**Authors:** Concetta Ragone, Carmen Manolio, Angela Mauriello, Beatrice Cavalluzzo, Franco M. Buonaguro, Maria Lina Tornesello, Maria Tagliamonte, Luigi Buonaguro

**Affiliations:** 1 Lab of Innovative Immunological Models, Istituto Nazionale per lo Studio e la Cura dei Tumori - IRCCS, “Fondazione Pascale”, Via Mariano Semmola, 52, 80131 Naples, Italy; 2 Molecular Biology and Viral Oncogenesis Unit, Istituto Nazionale per lo Studio e la Cura dei Tumori - IRCCS “Fond G. Pascale”, Via Mariano Semmola, 52, 80131 Naples, Italy

**Keywords:** Tumor associated antigens, Microbiota-derived antigens, Molecular mimicry, Cross-reacting T cells

## Abstract

**Background:**

The gut microbiota profile is unique for each individual and are composed by different bacteria species according to individual birth-to-infant transitions. In the last years, the local and systemic effects of microbiota on cancer onset, progression and response to treatments, such as immunotherapies, has been extensively described. Here we offer a new perspective, proposing a role for the microbiota based on the molecular mimicry of tumor associated antigens by microbiome-associated antigens.

**Methods:**

In the present study we looked for homology between published TAAs and non-self microbiota-derived epitopes. Blast search for sequence homology was combined with extensive bioinformatics analyses.

**Results:**

Several evidences for homology between TAAs and microbiota-derived antigens have been found. Strikingly, three cases of 100% homology between the paired sequences has been identified. The predicted average affinity to HLA molecules of microbiota-derived antigens is very high (< 100 nM). The structural conformation of the microbiota-derived epitopes is, in general, highly similar to the corresponding TAA. In some cases, it is identical and contact areas with both HLA and TCR chains are indistinguishable. Moreover, the spatial conformation of TCR-facing residues can be identical in paired TAA and microbiota-derived epitopes, with exactly the same values of planar as well as dihedral angles.

**Conclusions:**

The data reported in the present study show for the first time the high homology in the linear sequence as well as in structure and conformation between TAAs and peptides derived from microbiota species of the Firmicutes and the Bacteroidetes phyla, which together account for 90% of gut microbiota. Cross-reacting CD8^+^ T cell responses are very likely induced. Therefore, the anti-microbiota T cell memory may turn out to be an anti-cancer T cell memory, able to control the growth of a cancer developed during the lifetime if the expressed TAA is similar to the microbiota epitope. This may ultimately represent a relevant selective advantage for cancer patients and may lead to a novel preventive anti-cancer vaccine strategy.

**Supplementary Information:**

The online version contains supplementary material available at 10.1186/s12967-022-03512-6.

## Introduction

The human gastrointestinal tract is colonized by approximately 10^14^ microbes equating to roughly 1000 times the number of cells and 10,000 times the DNA content of the human body [1].

Gut microbiota include several species of microorganisms but the *Firmicutes* and *Bacteroidetes* phyla account for 90% of the total [2].

In addition, type of delivery [3], methods of milk feeding [4] and weaning period [5] have a major impact on the microbiota composition in the first year of life.

The child’s gut microbiota composition and diversity reaches the adulthood characteristics at approximately three years of age as consequence of genetics, environment, diet, lifestyle, and gut physiology [6]. Several factors may induce variations in the gut microbiota during the life, including lifestyle, dietary and cultural habits [7].

Gut bacteria regulate food digestion along the gastrointestinal tract and prevent bacteria invasion by maintaining the intestinal epithelium integrity [8]. In addition, they have been shown to regulate the development, homeostasis, and function of innate and adaptive immunity [9].

Besides the physiological role, the microbiota have a role in human diseases [10, 11] and influence both tumor development and treatment response [12–19]. In particular, specific microbiota populations have been show to affect cancer genesis and development via the production of selected metabolites [20]. Indeed, while components such as LPS or MPL activate T cell-mediated anti-cancer responses [21], the bacterial virulence factor Fap2 can inhibit Natural Killers [22]. Moreover, the polysaccharide A and TLR signaling to dendritic cells may drive the Tregs development [23, 24] as well as segmented filamentous bacteria (SFB) may induce Th17 cells [25].

In addition to such “generic” mechanisms of modulation, the impact of the gut microbiota on the anti-cancer immune response can be driven by a “molecular mimicry” between bacteria and tumor associated antigens.

The gut microbiome encodes over 3 million genes as whole, whereas the entire human genome consists of approximately 23,000 genes [7]. Therefore, the probability of homology between microbiota and human antigens is high, resulting in an overlapping peptidome representation. Highly similar epitopes can be targeted by the same CD8^+^ T cell receptor (TCR), given that a single TCR is cross-reactive recognizing at least 10^6^ different MHC-bound peptides [26, 27]. Indeed, the epitope binds to the HLA molecule with specific residues in fixed positions along the sequence (anchor residues) and only the central residues are exposed for recognition by the TCR (http://www.cbs.dtu.dk/services/NetMHC/logos.php) [28, 29]. Therefore, two unrelated antigens sharing the same TCR-facing central residues, or showing conservative variations at those positions, are very likely recognized by the same TCRs if the structural conformation of the entire epitope is saved.

Based on this assumption, we have recently shown that TAAs (https://caped.icp.ucl.ac.be/Peptide/list) share sequence homology to viral sequences [30]. This would suggest that viral antigens might elicit memory CD8^+^ T cells cross-reacting with tumor antigens, able to control the growth of a cancer lesion, if expressing a TAA similar to the viral epitope. This may ultimately represent a relevant selective advantage for cancer patients and may lead to a novel preventive anti-cancer vaccine strategy [31–33].

Here we report that significant sequence and conformational homology do exist also between TAAs and peptides derived from microbiota species of the *Firmicutes* and *Bacteroidetes* phyla. This suggests a much broader molecular mimicry between cancer and pathogens antigens with high potential cross-reactive T cell response and impact on tumor progression.

## Materials and methods

### Epitope prediction analysis.

All the peptides selected in the study were predicted with the NetMHCpan 4.1 and the NetMHCstabpan 1.0 predictive algorithms (https://services.healthtech.dtu.dk/service.php?NetMHCpan-4.1; https://services.healthtech.dtu.dk/service.php?NetMHCstabpan-1.0).

The peptides deposited at the cancer antigenic peptide database (https://caped.icp.ucl.ac.be/Peptide/list) were used to interrogate NetMHCpan 4.1 tool. Nanomer peptides for the MHC class I HLA-A* 0201 and 2402 alleles (http://www.allelefrequencies.net) have been selected with a predicted affinity value < 100 nM (Strong Binders, SB).

Likewise, microbiota nanomer peptides identified by the BLAST homology search were used to interrogate NetMHCstabpan 1.0 tool for the four most prevalent MHC class I HLA-A*0201 and 2402 alleles. Strong binder peptides were selected with a predicted affinity value < 100 nM and stability > 1 h.

### BLAST homology search

The TAAs selected as SB according to NetMHCpan 4.1 prediction tool have been submitted to BLAST for a protein homology search against Firmicutes (taxid:1239) and Bacteroidetes (taxid:976) sequences within the non-redundant protein sequences database (https://blast.ncbi.nlm.nih.gov/Blast.cgi). Homologous microbiota protein sequences have been extracted from the protein database of the National Center for Biotechnology Information (NCBI) (https://www.ncbi.nlm.nih.gov/) and epitope prediction has been performed with the NetMHCstabpan 1.0 tool.

### Epitope modelling and molecular docking

Specific reference structures were selected from the protein data bank PBD (https://www.rcsb.org) for the HLA-A*02:01 and HLA-A*24:02 molecules. The 1AO7 complex was selected which includes the structure of the HTLV-I LLFGYPVYV epitope crystallized with the HLA-A*02:01 molecule, the β2 microglobulin, the α and β chains of the TCR (PDB https://www.rcsb.org/structure/1AO7). The 7JYV complex was selected which includes the structure of the influenza YFSPIRVTF epitope crystallized with the HLA-A*24:02 molecule (https://www.rcsb.org/structure/7JYV). The PyMol software (PyMol Molecular graphics system, version 1.8.6.2) was used to modify the epitope sequences into the peptides analyzed in the present study. The Molsoft Mol Browser (version 3.8-7d) was used to generate the epitope modelling and molecular docking as well as to calculate the inter-atom planar and dihedral angles.

## Results

### Blast search for homology between Tumor and microbiota antigens

Peptides from the cancer antigenic peptide database, predicted as strong binders (SB) to the most frequent MHC class I alleles were selected, as previously described [30].

**Fig. 1:**
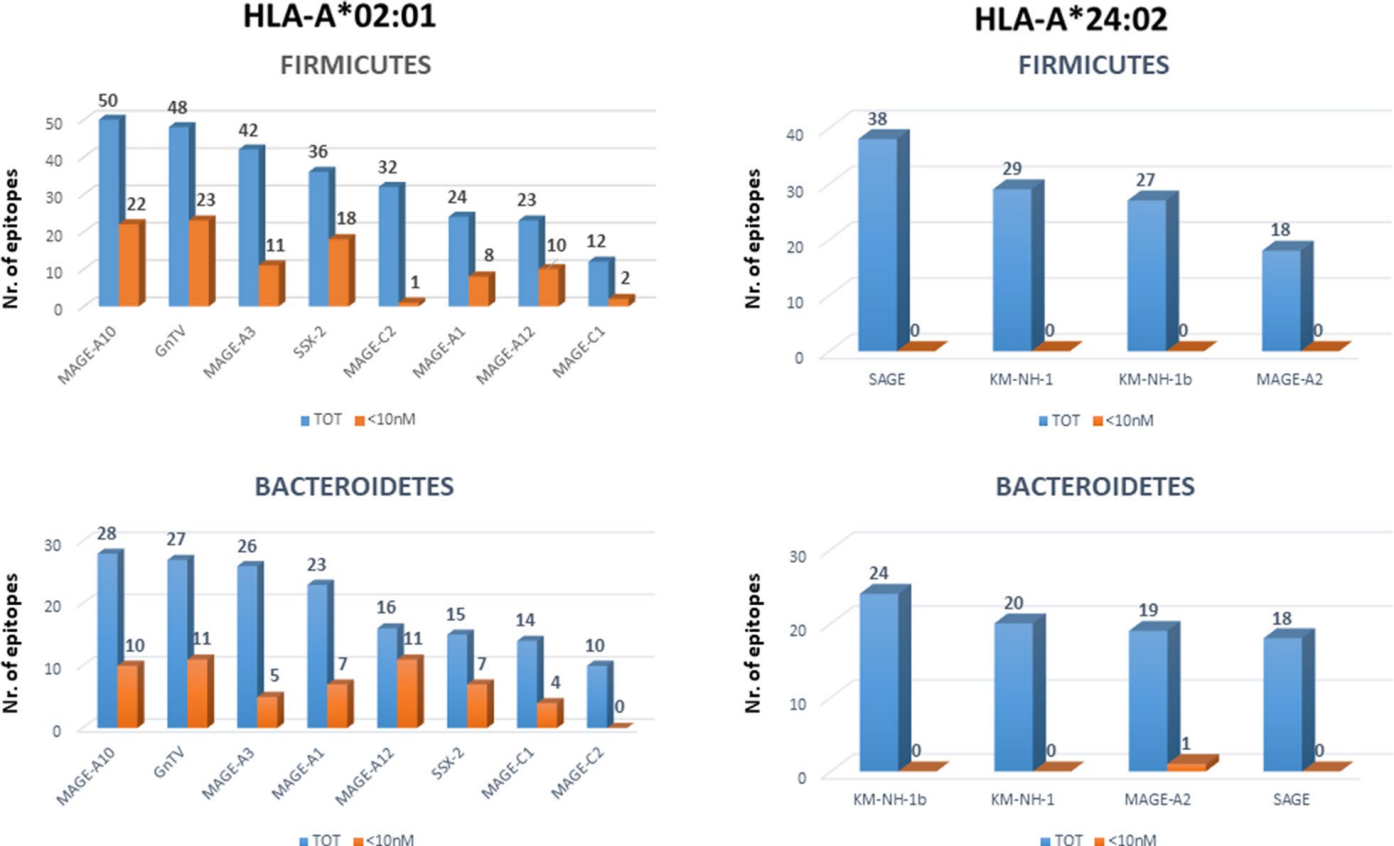
Microbiota-derived peptides homologous to TAA. The number of peptides derived from Firmicutes and Bacteroidetes phyla with homology to individual TAA are indicated. The blue bars indicate the total number of peptides; the orange bars indicate the ones with high affinity to the HLA molecules (< 10 nM)

Subsequently they were subjected to global protein BLAST against the bacteria sequences of the *Firmicutes* and *Bacteroidetes* phyla within the GeneBank non-redundant protein database. The search returned a large number of HLA-A*0201 and 2402 restricted bacterial sequences sharing homology with selected TAAs and the majority of such homology was found for the HLA-A*0201. Indeed, 267 and 159 homologous peptides were identified for Firmicutes and Bacteroidetes, respectively, for HLA-A*02:01. Of these, 95 and 55 were identified with a high affinity value < 10 nM for Firmicutes and Bacteroidetes, respectively. The MAGE-A10 was the TAA with the highest numbers of homologous peptides for both Firmicutes and Bacteroidetes (e.g. 50 and 28) while the MAGE-C1 and MAGE-C2 were the TAA with the lowest numbers of homologous peptides for Firmicutes and Bacteroidetes (e.g. 12 and 10), respectively. On the contrary, 112 and 81 homologous peptides were identified for Firmicutes and Bacteroidetes, respectively, for HLA-A*24:02. Of these, none and only 1 were identified with a high affinity value < 10 nM for Firmicutes and Bacteroidetes, respectively. The SAGE and the KM-NH-1b were the TAA with the highest numbers of homologous peptides for Firmicutes and Bacteroidetes (e.g. 38 and 24), respectively; while the MAGE-A2 and SAGE were the TAA with the lowest numbers of homologous peptides for Firmicutes and Bacteroidetes (e.g. 18 and 18), respectively (Fig. 1).

### Sequence homology between tumor and microbiota antigens

**Fig. 2:**
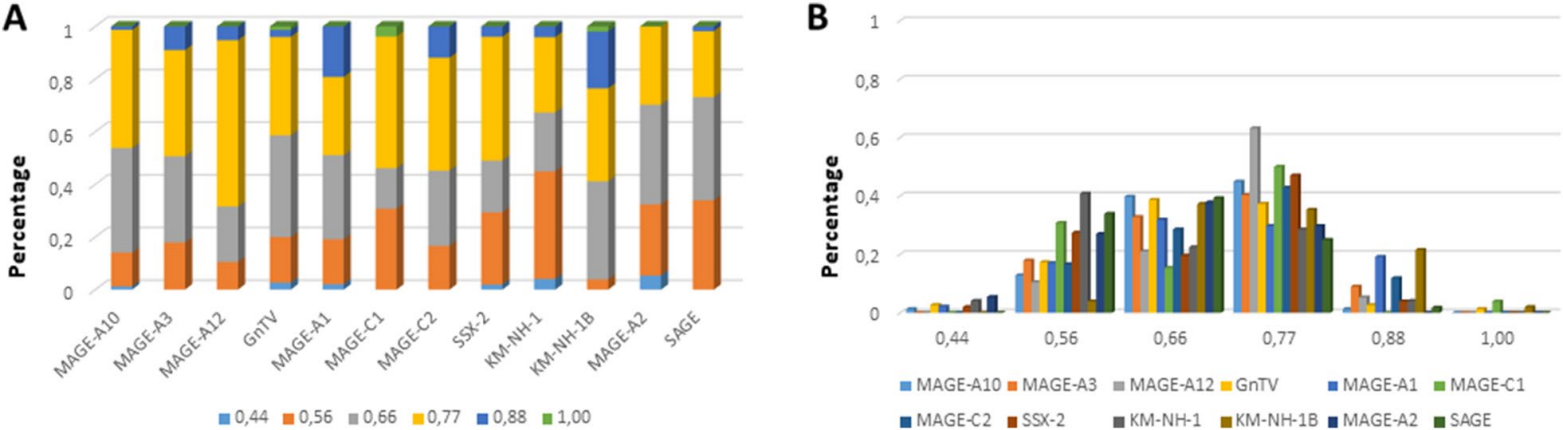
Homology between aminoacid residues in paired TAA and microbiota-derived peptides. The number of identical aminoacid residues in the paired nanomer TAA and microbiota-derived peptides is shown as percentage over the 9 aminoacids. The results are shown for each individual TAA (**A**) and for each percentage value (**B**)

The alignment of peptide sequences showed high levels of homology between TAAs and peptides from bacteria species of the two phyla. Indeed, the vast majority of microbiota-derived peptides (69.7% on average, minimum 51% for KM-NH-1—maximum 84.6% for MAGE-A10) showed 6 or 7 residues along the sequence identical to the corresponding TAA (Table 1; Fig. 2).

**Table 1:**
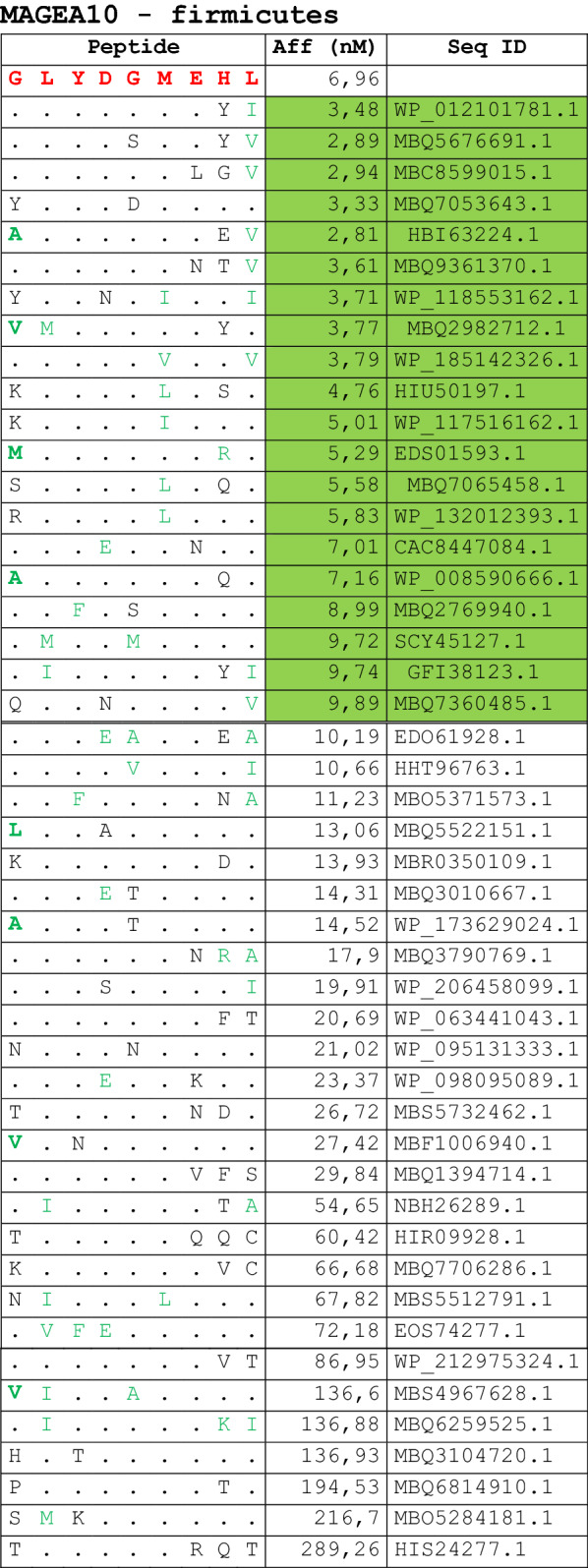

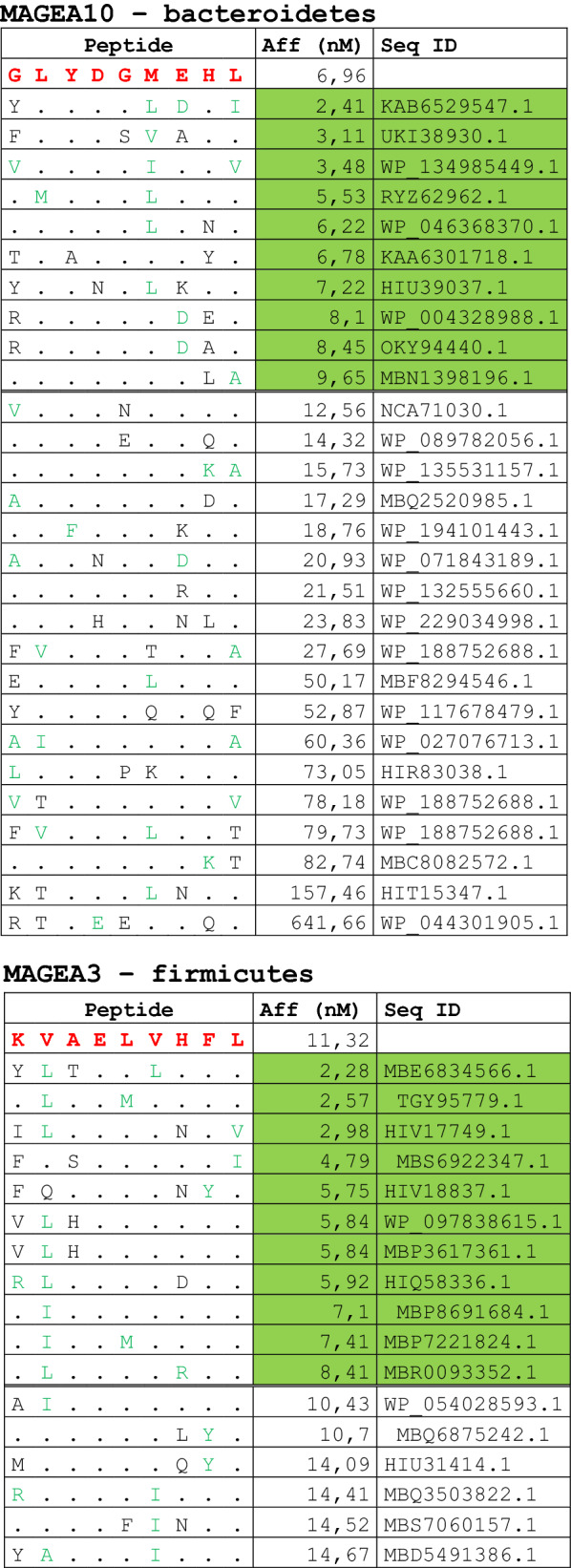

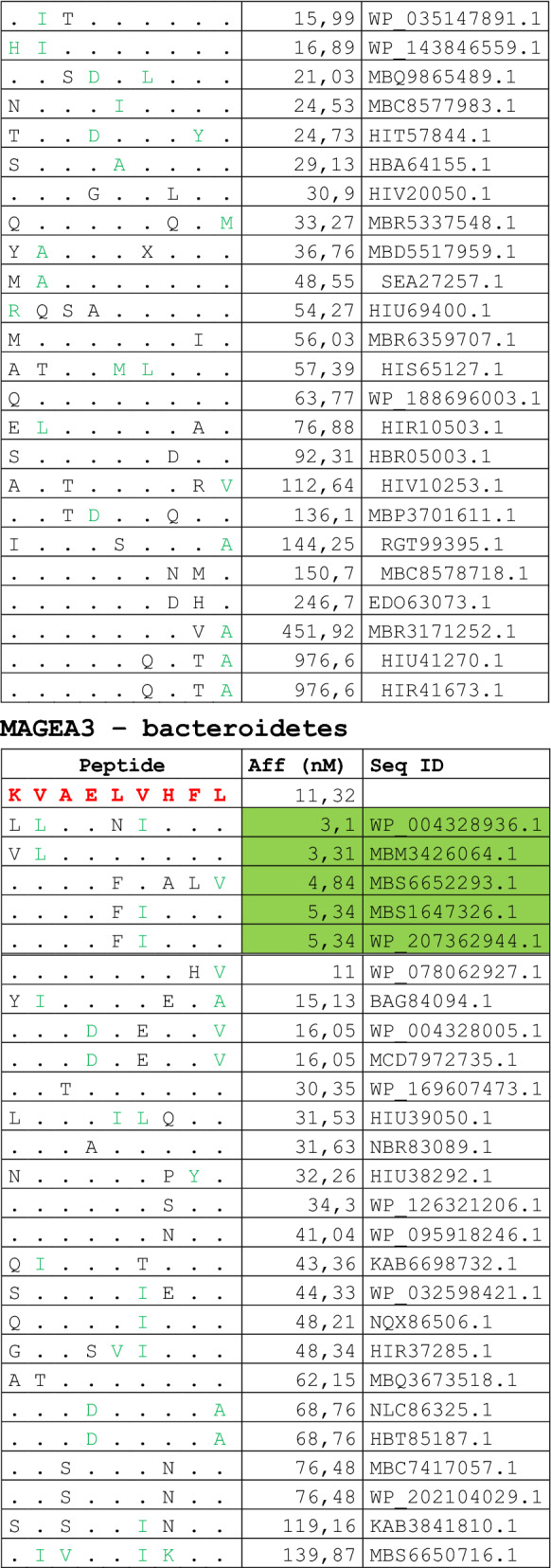

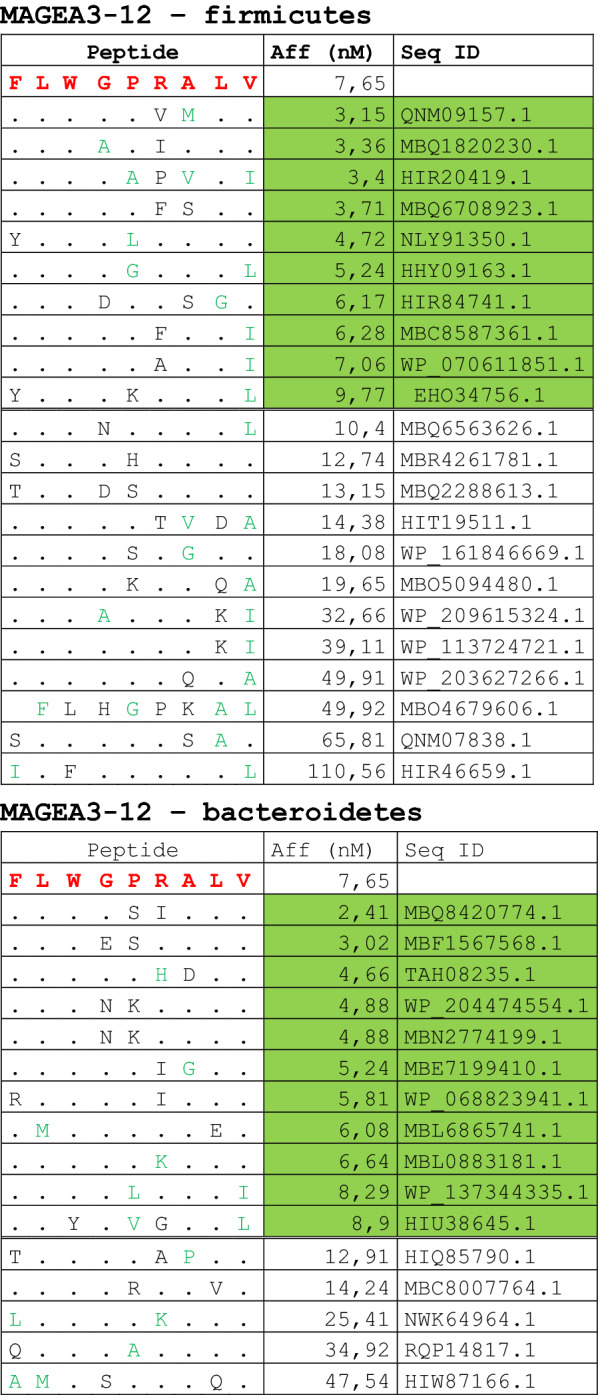

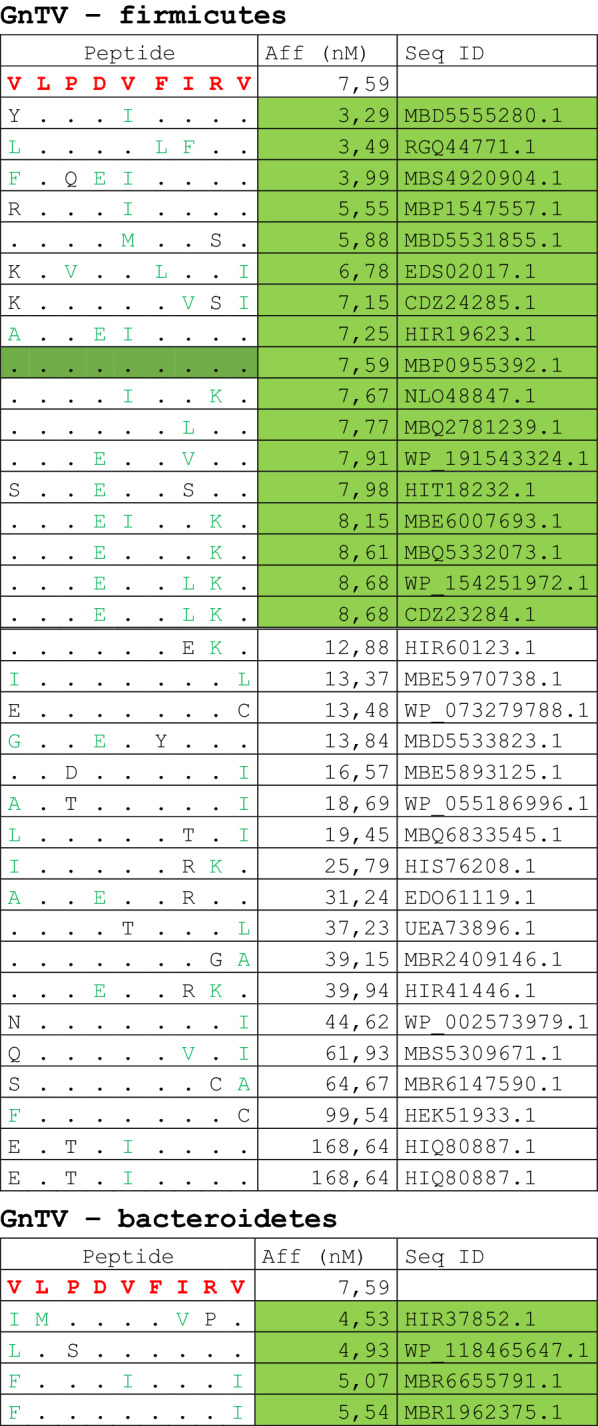

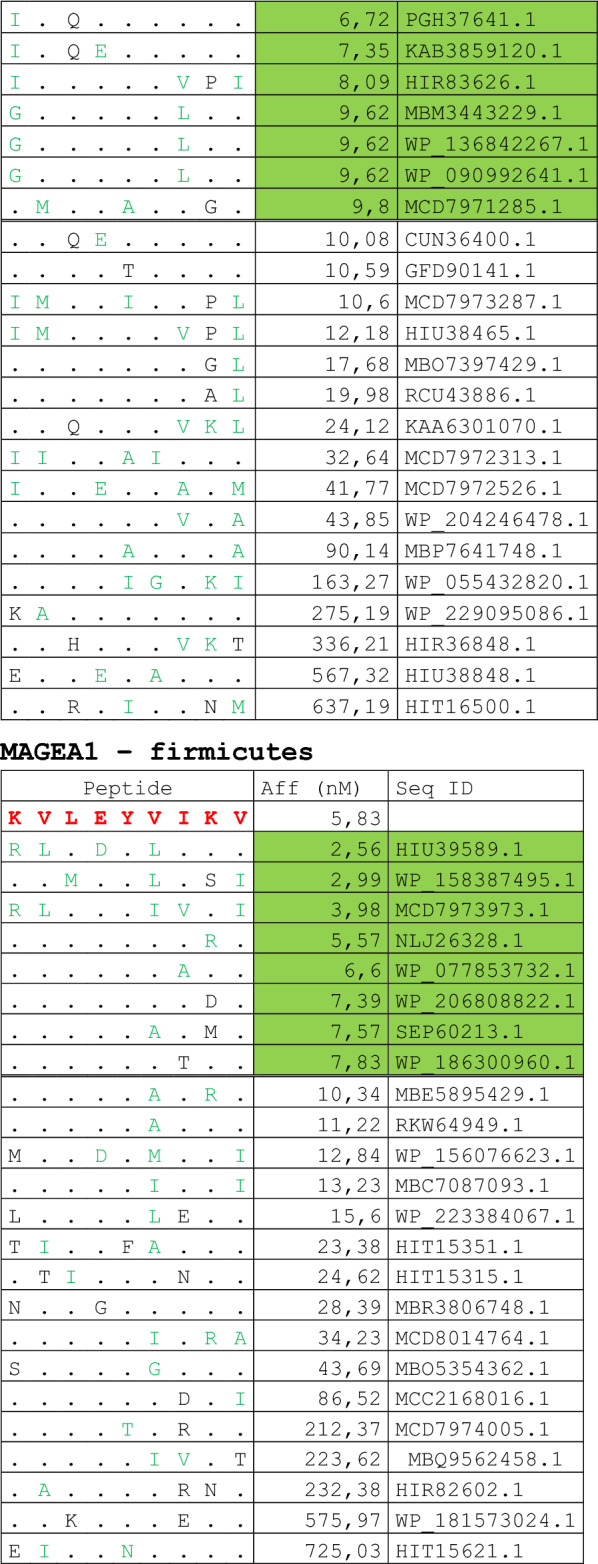

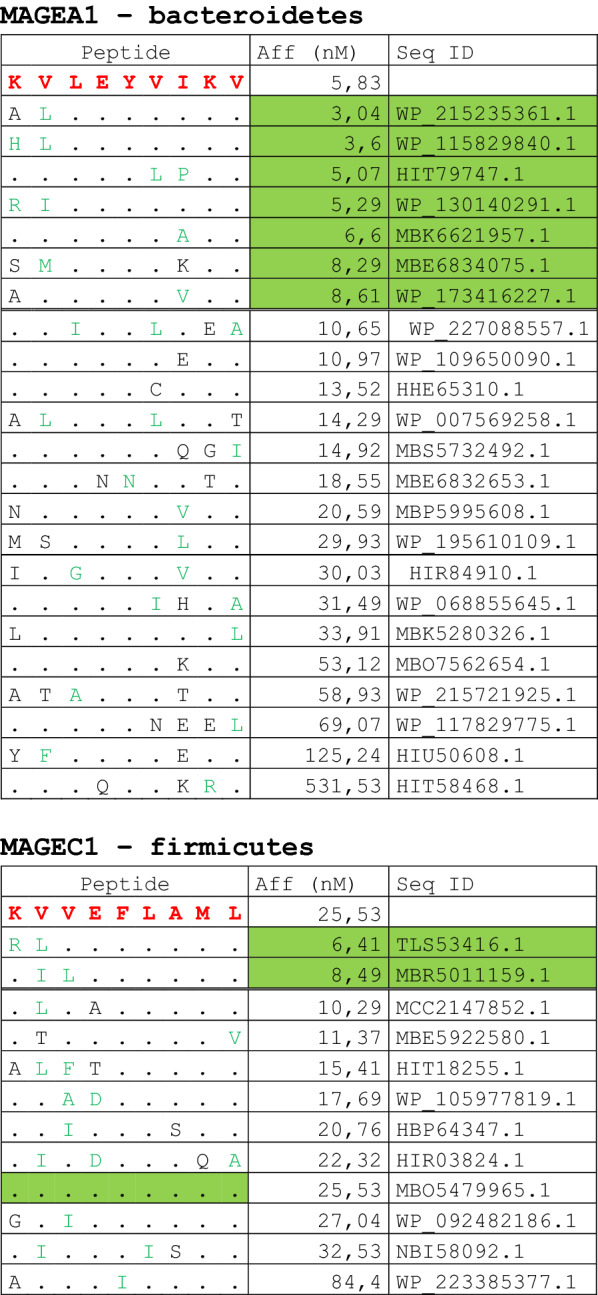

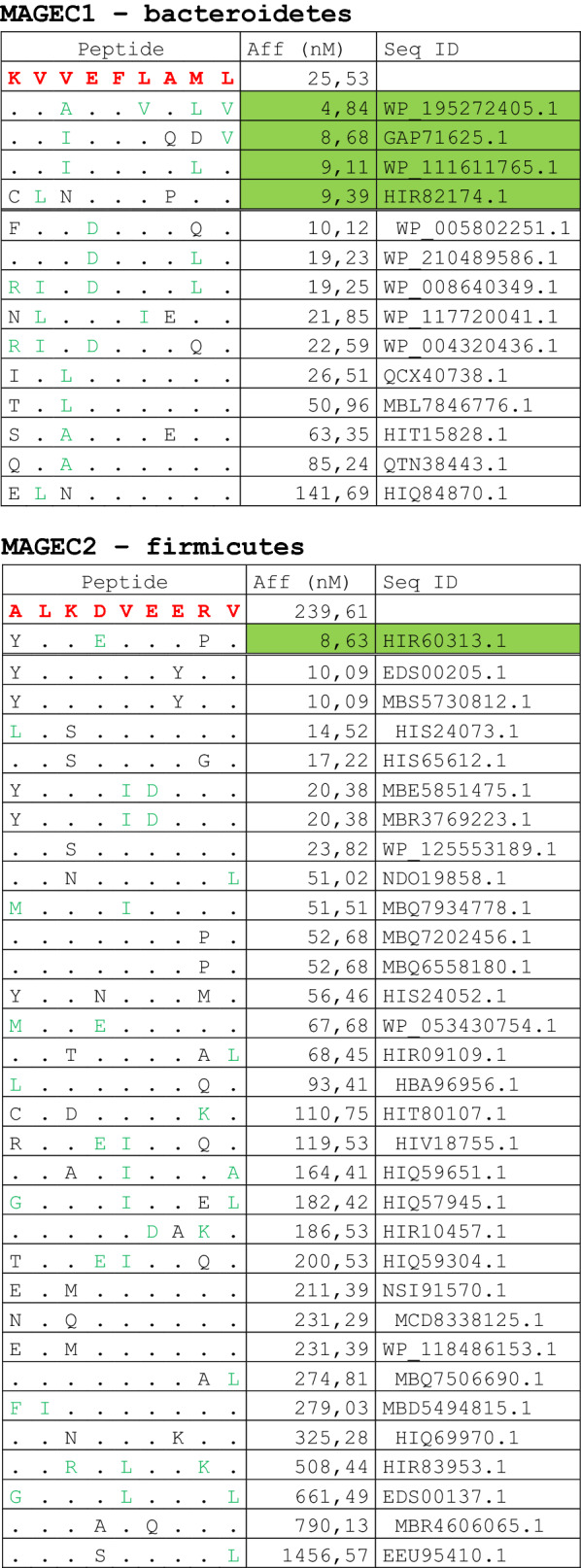

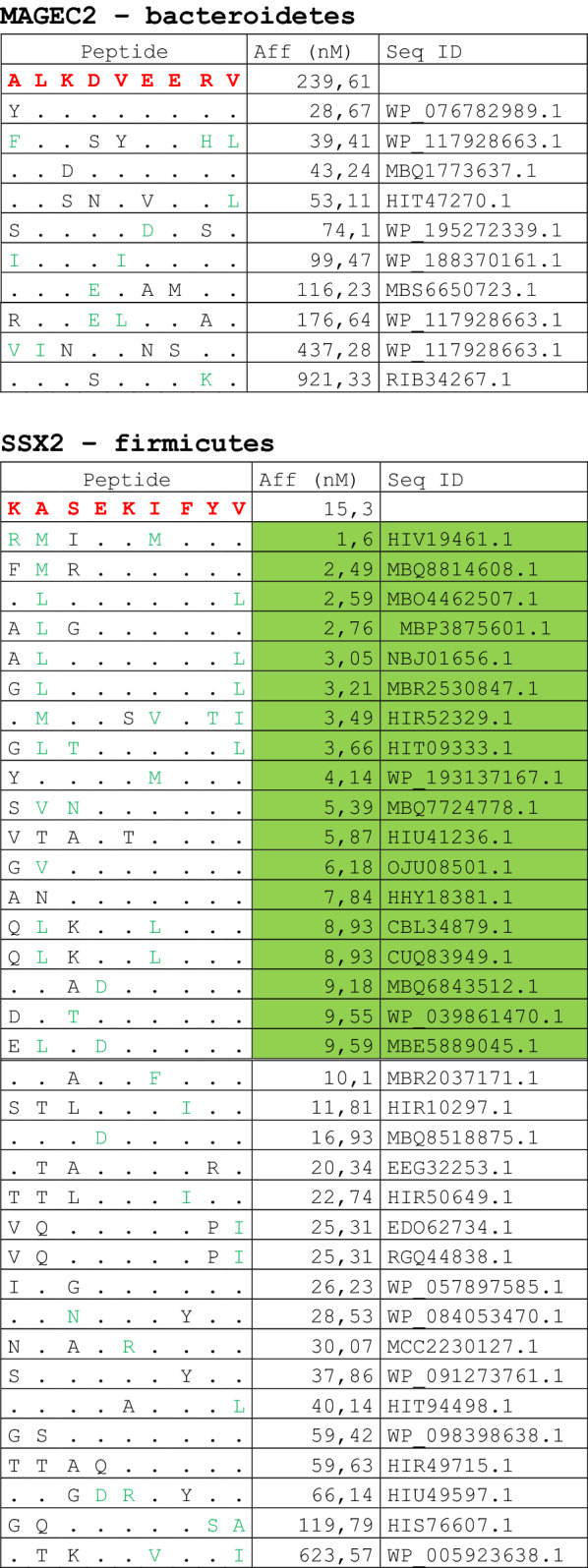

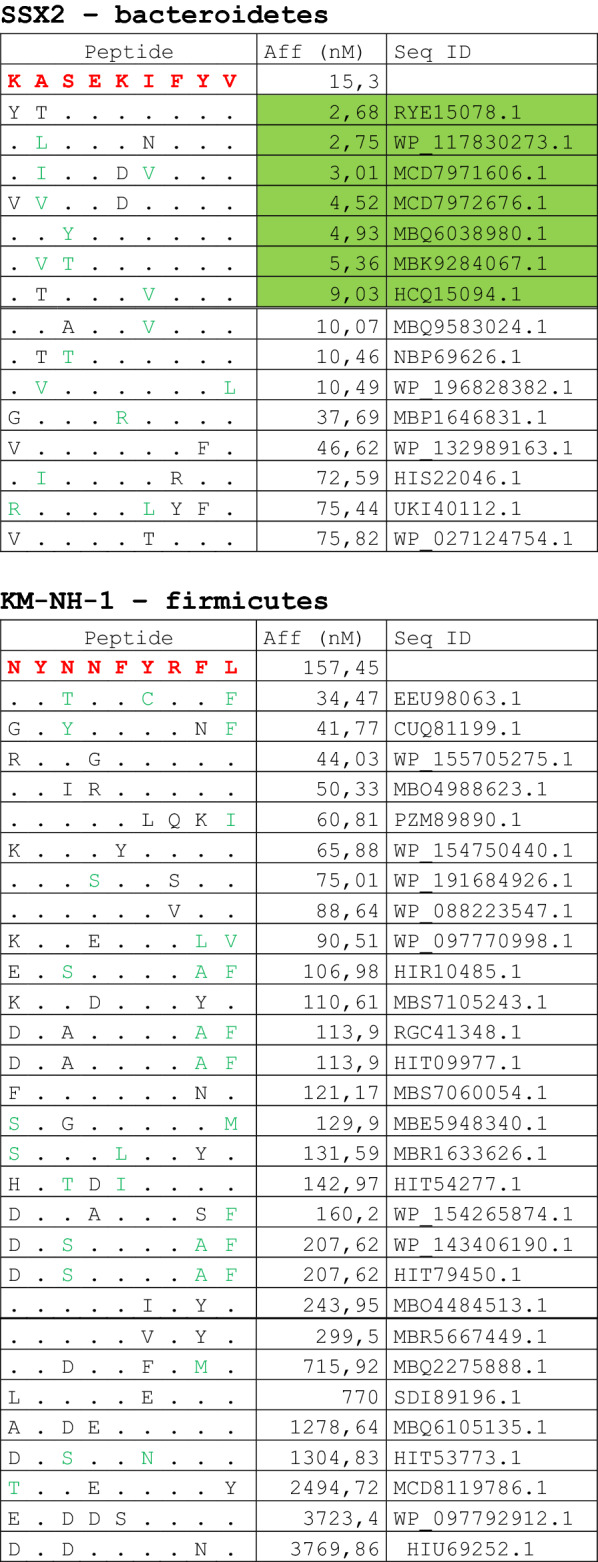

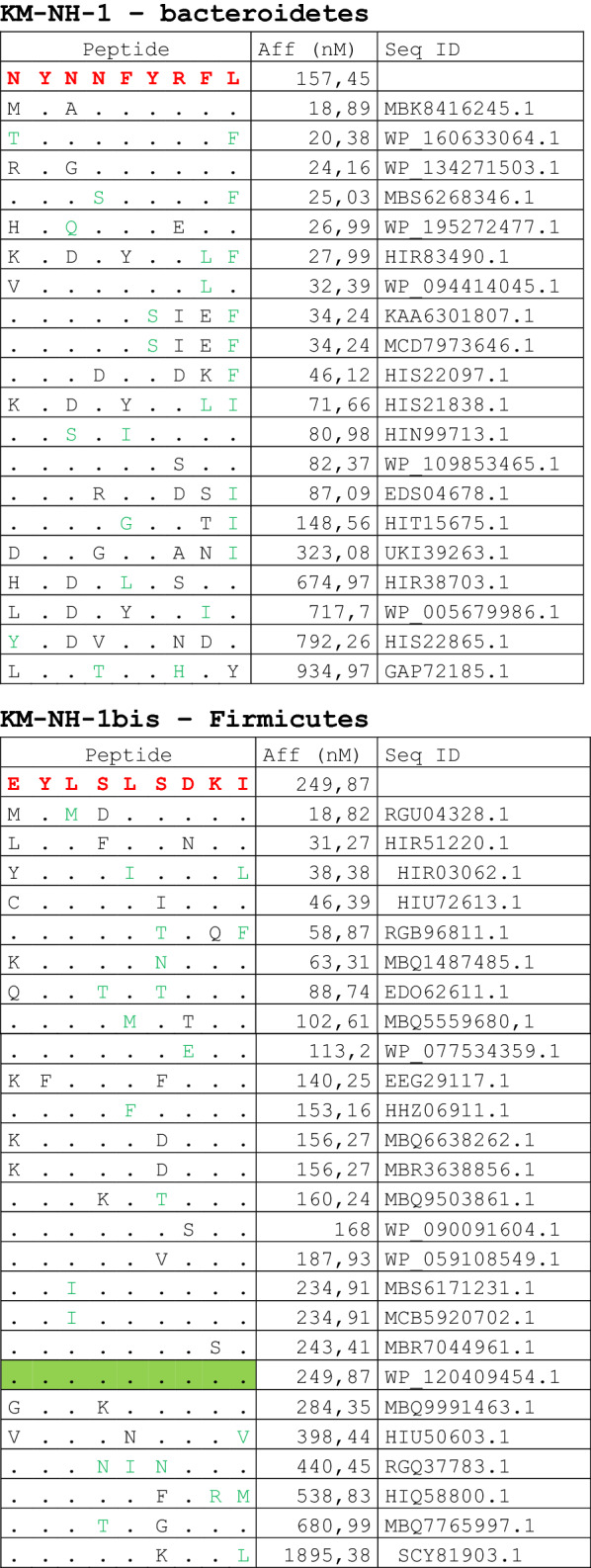

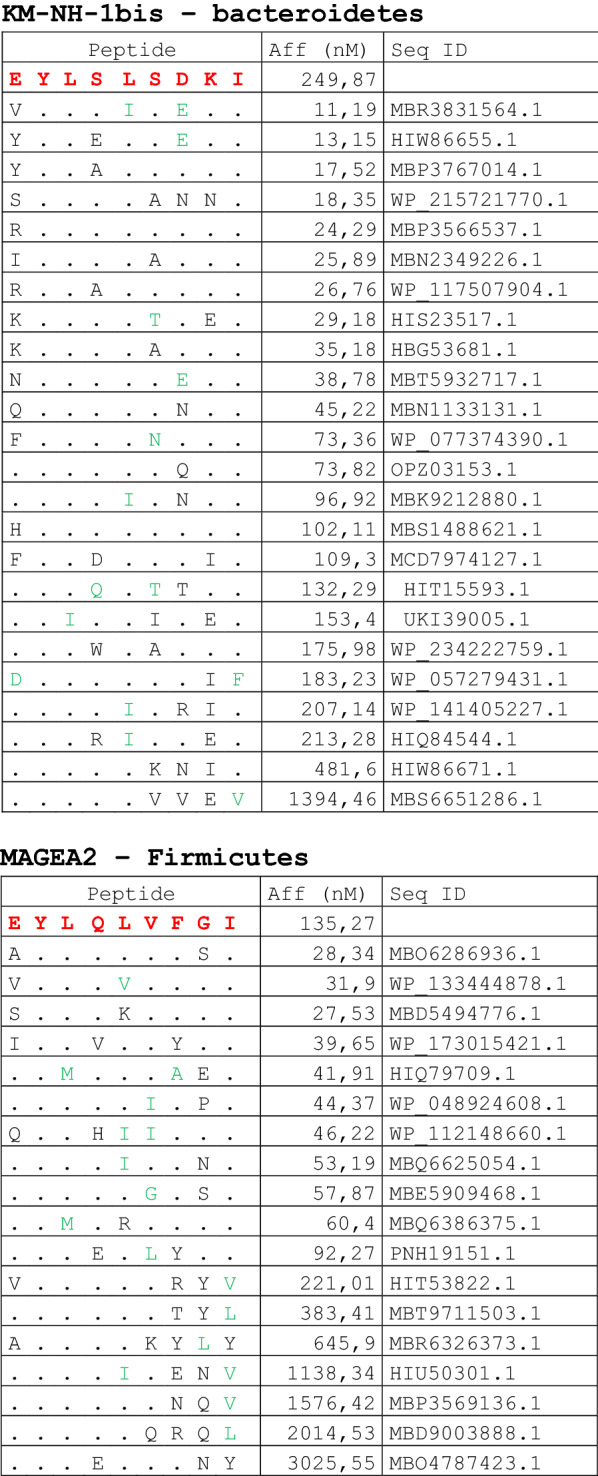

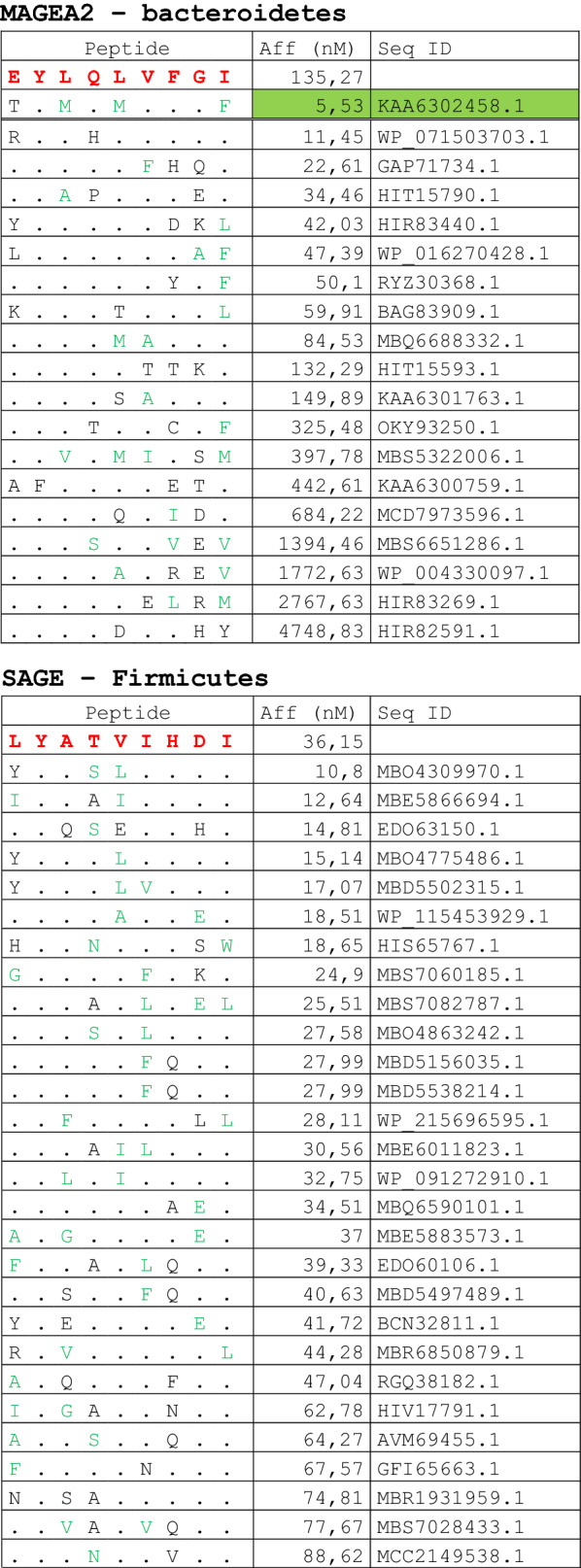

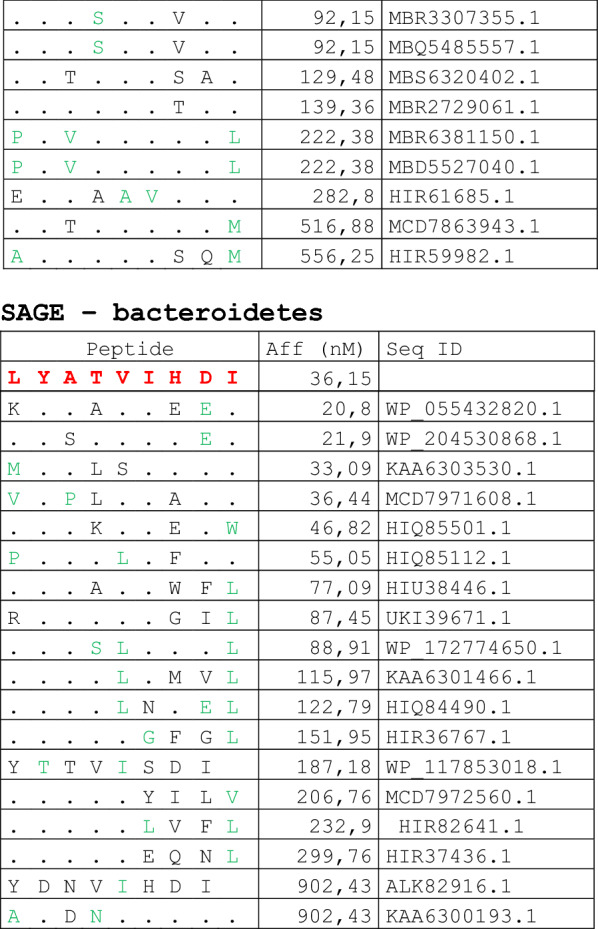
Alignment of microbiota-derived peptides and corresponding TAA

Dots = Identical residues, Green letters = conservative residues, Black letters = non-conservative residues. The green shades indicate indicated peptides with high affinity to HLA (<10 nM)

**Fig. 3:**
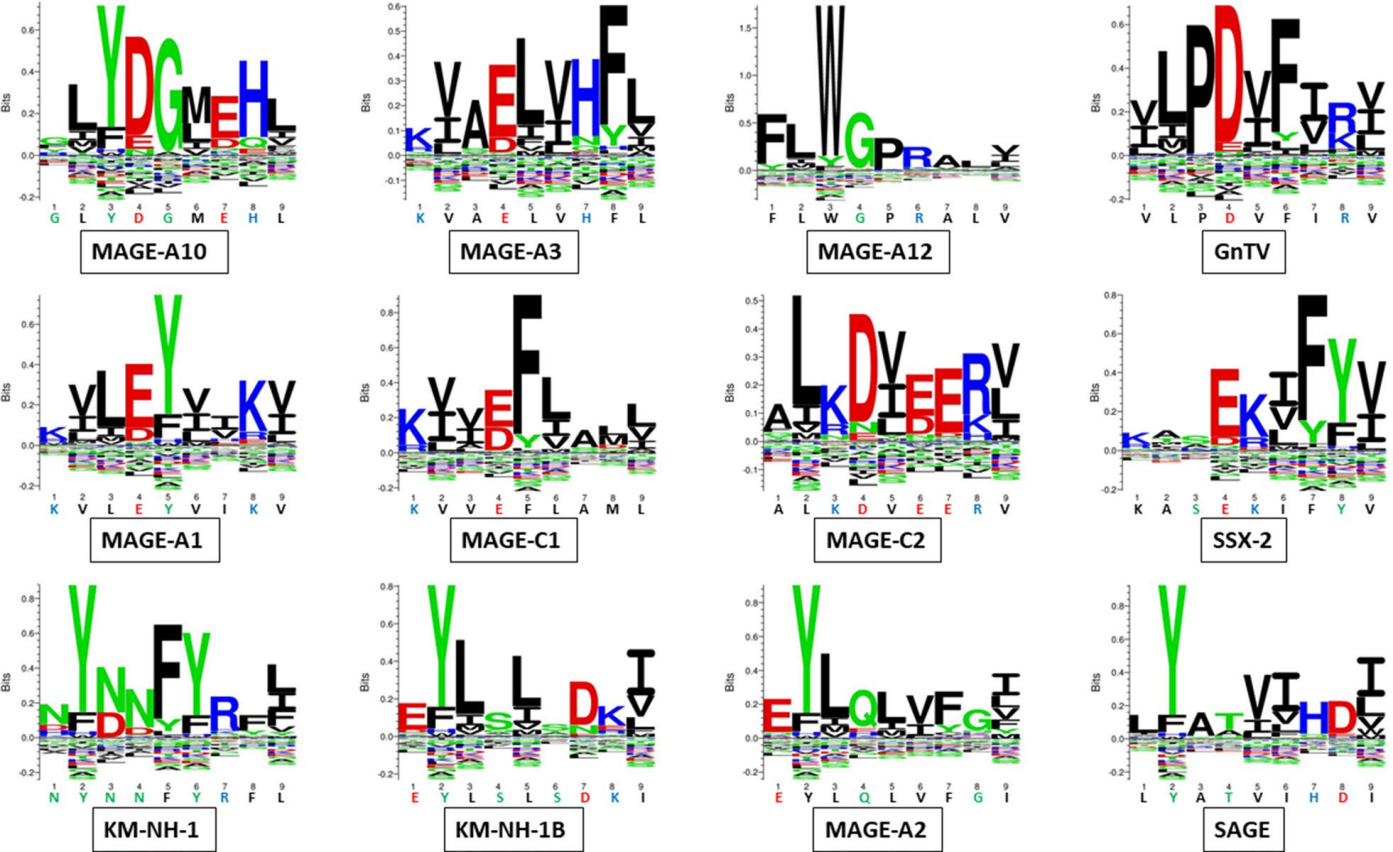
Sequence Logos of microbiota-derived epitopes. Amino acid sequences from all the microbiota-derived epitopes with homology to each TAA were piled up to build sequence logos. The height of the stack indicates the sequence conservation at that position, while the height of symbols within the stack indicates the relative frequency of each amino or nucleic acid at that position (https://services.healthtech.dtu.dk/service.php?Seq2Logo-2.0). Different colours indicate different class of aminoacid

Strikingly, three microbiota-derived peptides showed a sequence identical to the corresponding TAA, the GnTV (Firmicutes Oscillospiraceae bacterium Seq ID MBP0955392.1), MAGE-C1 (Firmicutes Clostridia bacterium Seq ID MBO5479965.1) and MK-NH-1 (Firmicutes Roseburia sp. 1XD42-69 Seq ID WP_120409454.1) (Table 1). To further confirm the high homology between the paired epitopes, the pattern of the microbiota-derived peptides showed that the most frequent amino acid residues at each position perfectly matched with those in the sequence of the corresponding TAA (Fig. 3).

The alignment of the TAAs with homologous bacterial peptides shows that the amino acid residues at each position are mostly identical or of the same class (conservative substitution) (Table 1).

**Fig. 4:**
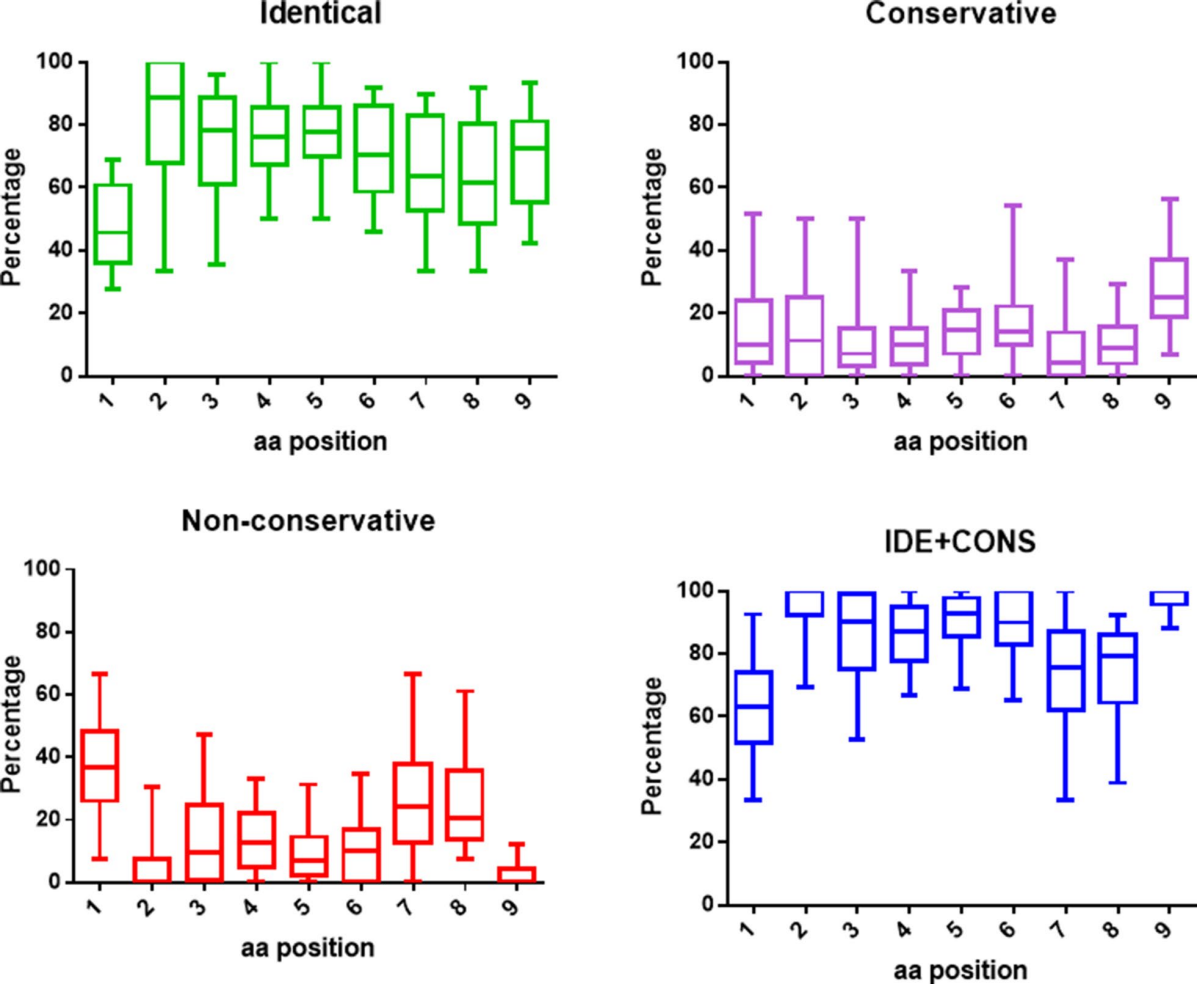
Comparison of amino acid residues between paired TAA and microbiota-derived peptides. The type of amino acid substitution was evaluated at each peptide position comparing the paired TAA and homologous peptides derived from microbiota. The type of substitutions at each position is represented as percentage of the total number of sequences

Indeed, the average percentage of identical residues at each position is 82.8%, ranging from 79.7% at position 3 to 89.5% at position 2. Summing up also the conservative substitutions, the average percentage of at each position is 93.6%, ranging from 86.7% at position 7 to 98.3% at position 9. Consequently, the average percentage of non-conservative substitutions at each position is only 6.39%, ranging from 1.6% at position 9 to 13.2% at position 7. In such analysis, position 1 represents an outlier with the far lowest average percentage of identical (48.2%) and identical + conservative substitutions (62.4%) and the far highest average percentage of non-conservative substitutions (37.6%) (Fig. 4). The percentage of different type of substitutions at each position for the microbiota-derived peptides significantly varies among TAAs but the trend is overall confirmed (Additional file 1: Fig. S1–4).

### Affinity to HLA molecules

**Fig. 5:**
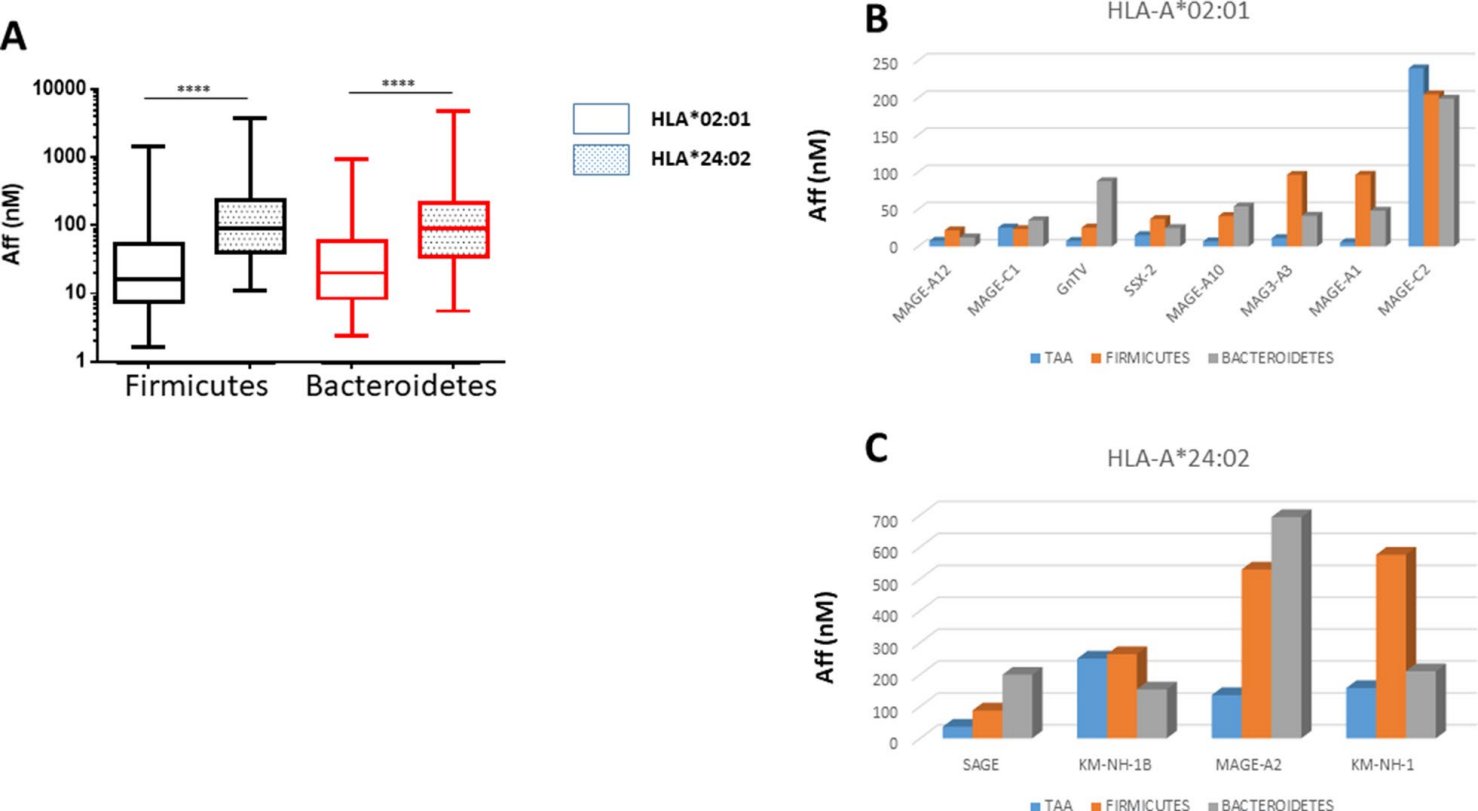
Affinity and stability of paired peptides. The affinity to HLA*02:01 (**A**) and HLA*24:02 (**B**) molecules were predicted by NetMHCstabpan for each TAA and paired bacterial peptides. The affinity to HLA molecules of all bacterial peptides derived from species of the Firmicutes and Bacterioidetes phyla are represented (**C**). The affinity values (Aff) are expressed in nanomolarity (nM)

The predicted affinity of the microbiota-derived peptides to the HLA-A molecules was, on average, very high. In particular, the average affinity was 145.1 nM and 128.5 nM when considering all peptides derived from Firmicutes and Bacteroidetes, respectively. Interestingly, the average affinity values were significantly higher for the Firmicutes and Bacteroidetes peptides restricted for HLA*02:01 (68.6 and 56.5 nM, respectively) than for HLA*24:02 (327.5 and 335.54 nM, respectively) (Fig. 5A). Considering each HLA*02:01-restricted TAA, the homologous microbiota-derived peptides showed an affinity ranging from 21.8 (MAGE-A12) to 204.8 (MAGE-C2) (Firmicutes) and from 12.2 (MAGE-A12) to 198.9 (MAGE-C2) (Bacteroidetes). Considering each HLA*24:02-restricted TAA, the homologous microbiota-derived peptides showed an affinity ranging from 87.4 (SAGE) to 575.8 (KM-NH-1) (Firmicutes) and from 153.4 (KM-NH-1B) to 693.3 (MAGE-A2) (Bacteroidetes) (Fig. 5B and C). In all cases, more than 80% of the microbiota-derived peptides showed a binding affinity lower than the average value and 20 – 50% of them showed a binding affinity lower than the corresponding TAA (Additional file 1: Fig. S5).

### Epitope modeling and molecular docking

In order to verify whether the predicted paired TAA and bacterial epitopes share similar conformation and contact residues with the HLA molecule, epitope modeling and molecular docking were performed taking advantage of crystallized structures deposited in PDB. In addition, the comparison of the contact residues to TCR chains was possible only for HLA-A*02:01 restricted epitopes, due to the lack of crystallized structures including both HLA and TCR for HLA-A*24:02 deposited in the PDB (Additional file 1: Fig. S6–S17).

**Fig. 6:**
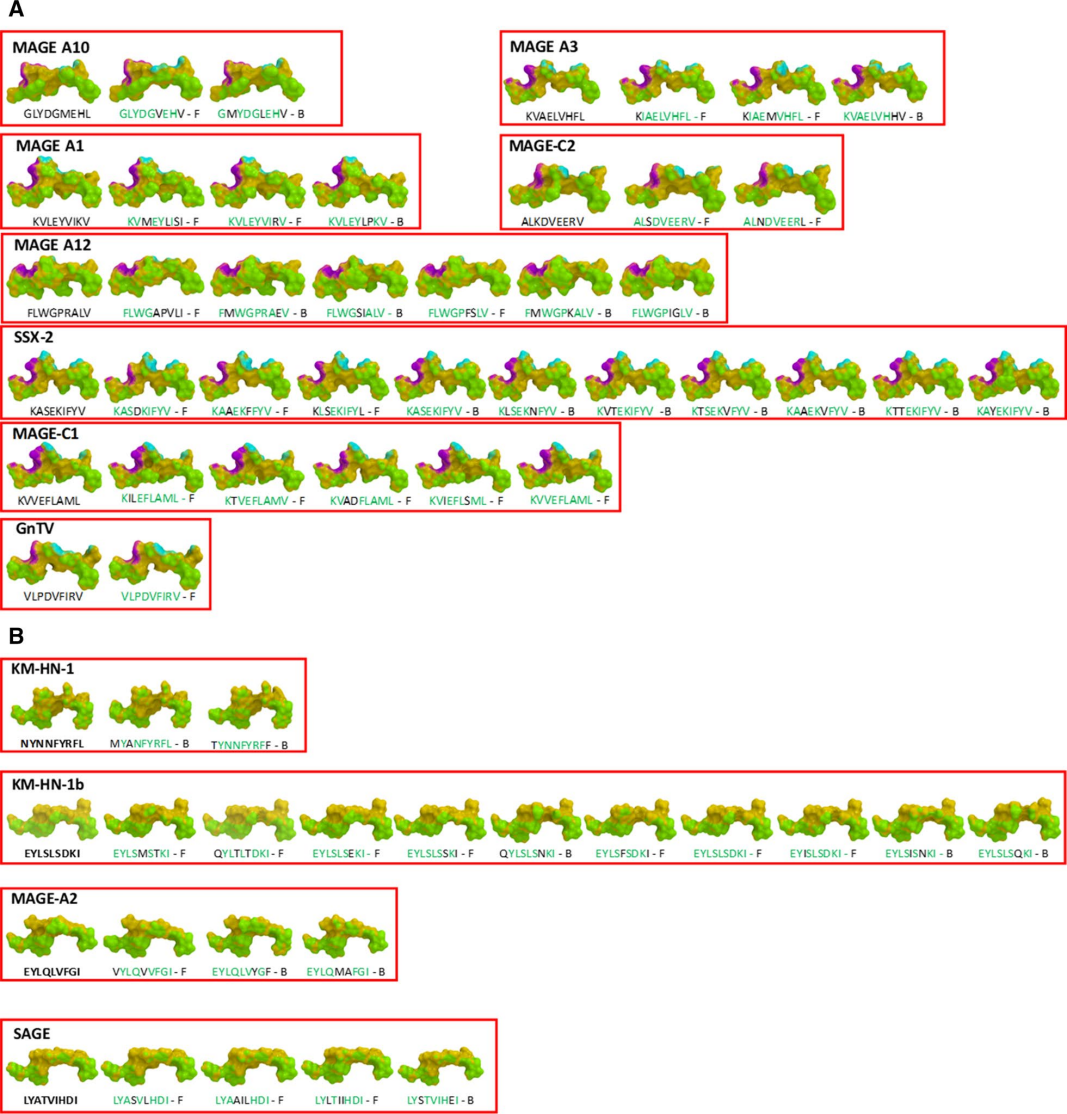
Predicted 3D conformation of TAA and microbiota-derived paired peptides. The surface conformation of the most similar paired TAA microbiota-derived peptides is shown. **A** HLA-A*02:01 and **B** HLA-A*24:02 restricted peptides are shown. Residues in the microbiota-derived epitopes (F = firmicutes; B = bacteroidetes) identical to the TAA sequences are indicated in green color. Green areas = contact points with HLA-A molecule; Magenta areas = contact points with TCR α chain; Light Blue = contact points with TCR β chain

Some of the peptide structures from the microbiota-derived epitopes show a pattern of contact with the HLA molecule and the TCR chains (for the HLA-A*02:01) strikingly similar to the corresponding TAA. Indeed, in some cases, the structures of paired TAA and microbiota-derived epitopes are indistinguishable (Fig. 6A and B).

**Fig. 7:**
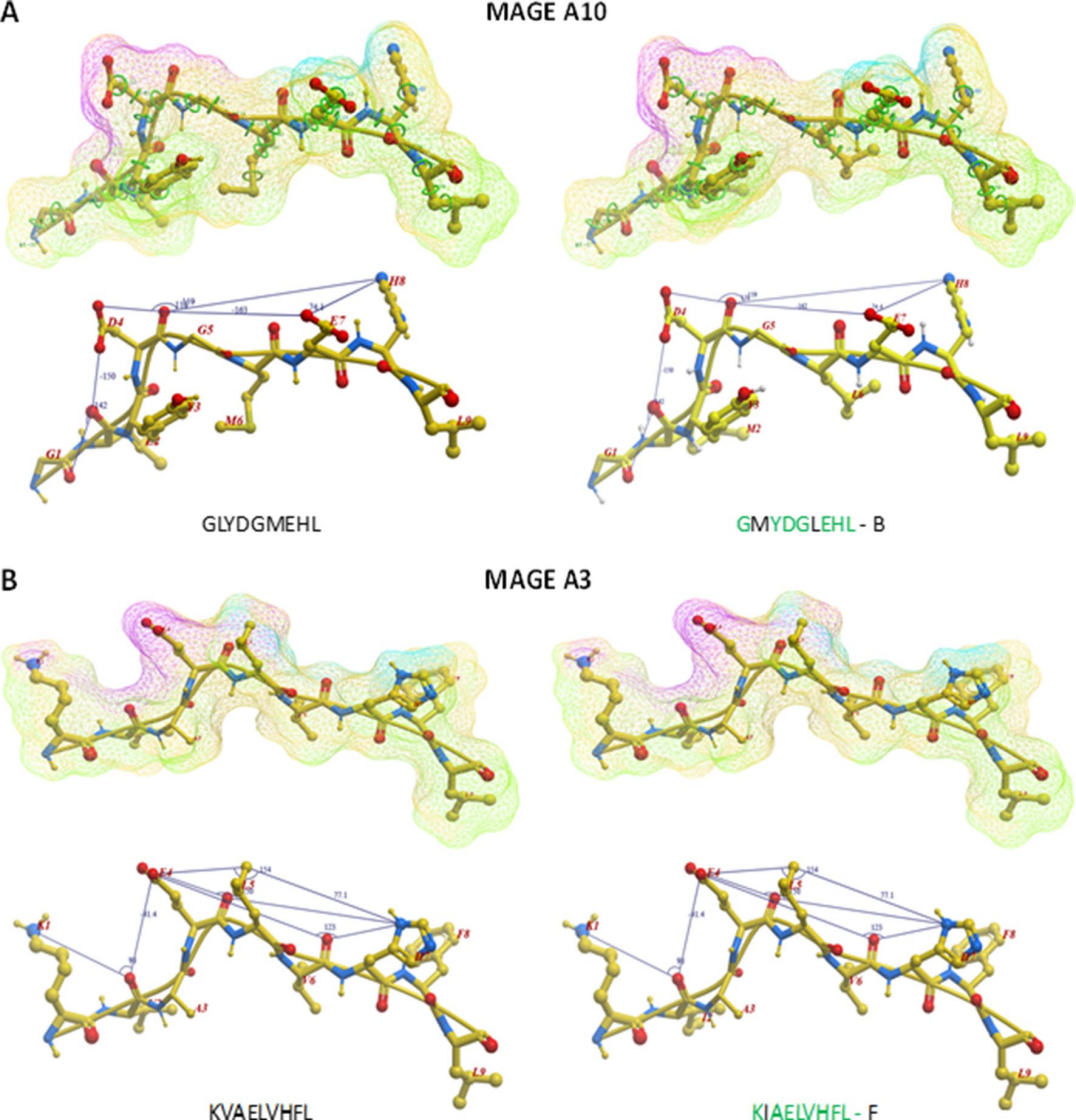
Structural predicted backbone conformation of TAA and bacterial paired peptides. The backbone conformation of the paired peptides bound to the HLA-A*02:01 molecule is shown. **A** Contact points on the surface of molecules are shown for MAGE-A10 and paired Bacteroidetes Chitinophagaceae bacterium. **B** Contact points on the surface of molecules are shown for MAGE-A3 and paired Firmicutes Sedimentibacter sp. Green areas = contact points with HLA molecule; Magenta areas = contact points with the TCR α chain; light blue areas = contact points with the TCR β chain. Values of the planar as well as dihedral angles between atoms of selected TCR-facing residues are shown in parallel

Additional information about the similarity between the paired TAA and microbiota-derived epitopes is provided by the peptide backbones. Indeed, considering the spatial position of the residues interacting with the TCR, it is possible to identify paired epitopes which share the same planar as well as dihedral angles (± 10%) between selected atoms of the amino acid residues in the same position of the peptide. Such analysis shows that, among paired TAA and microbiota-derived epitopes with highly similar structures, it is possible to identify selected pairs with 100% identical angle values, especially in HLA-A*02:01 epitopes (Additional file 1: Fig. S18–24). As example, the GLYDGMEHL MAGE-A10 and the GMYDGLEHV (Bacteroidetes Chitinophagaceae bacterium) peptides with M to L substitution in position 2 and an L to M substitution in position 6 shows identical spatial conformation of TCR-facing residues (Fig. 7A). Same result is observed for the KVAELVHFL MAGE-A3 and the KIAELVHFL (Firmicutes Sedimentibacter sp.) peptides with a single I to V substitution in position 2 (Fig. 7B).

## Discussion

The data reported in the present study show for the first time the high homology in the linear sequence as well as in structure and conformation between TAAs and peptides derived from microbiota species of the Firmicutes and the Bacteriodetes phyla, which together account for 90% of gut microbiota.

The number of homologous peptides with high affinity (< 10 nM) to HLA-A*02:01 molecule indicates that the molecular mimicry between TAA and microbiota-derived epitopes is not anecdotal and suggest a potential relevance in cross-reacting T cell response. In particular, about 70% of the paired epitopes show 6–7 identical residues along the 9-aa peptide sequence, 7% of them shows 8 identical residues and three of them show an identical sequence. Such long stretches of identical amino acids in a nonamer sequence suggest a great biological relevance given that a random event of an identical stretch of 6–9 amino acid in a nonamer sequence has an extremely low probability to occur (1.56 × 10^–8^–1.95 × 10^–12^). To further confirm the biological relevance of such a molecular mimicry, the consensus of the microbiota-derived epitopes always perfectly matches with the sequence of the corresponding TAA frequent residues. Moreover, the amino acid substitutions at each position are mostly conservative, confirming that the replacement does not significantly impact on the charge and conformation of the peptide structure.

The average predicted affinity of the microbiota-derived epitopes to the HLA-A molecules is extremely high, especially for the HLA-A*02:01 molecule (< 100 nm) and in about 50% of cases this is even higher than the corresponding TAA. According to our previous studies, such affinity values have a 100% correspondence to experimentally validated binding to HLA molecules. Therefore, this supports the concept that such microbiota-derived epitopes are indeed efficiently presented on the surface of cells to elicit a T cell response. Finally, the structural conformation of the microbiota-derived epitopes is, in general, highly similar to the corresponding TAA. In some cases, it is identical and contact areas with both HLA and TCR chains are indistinguishable. The spatial conformation of TCR-facing residues can be identical in paired TAA and microbiota-derived epitopes, with exactly the same values of planar as well as dihedral angles. This confirms that specific amino acid substitutions in the linear sequence do not affect the peptides structure and conformation which can be recognized by cross-reacting T cells. In support to this hypothesis, we have recently shown that CD8^+^ T cells cross-react with TAAs and peptides derived from viruses, sharing the same level of homology observed in the present study [24].

The biological relevance of the findings herein described is that, depending on the stage of the human life when the individuals will encounter the microbiota species, such homology may represent a favorable or unfavorable factor. Indeed, if the microbiota species have colonized the gastrointestinal tract in the first few months of life, the immune system could recognize the derived peptides as self-antigens and the specific T cell clones would be removed. In this case, a tumor lesion presenting a homologous TAA would have a selective advantage for establishing and progressing with a poor prognosis.

On the contrary, if the microbiota species have colonized the gastrointestinal tract later during life, the immune system could recognize the derived peptides as non-self-antigens and specific memory T cell clones would be established. In this case, as for a preventive vaccine, a tumor lesion presenting a homologous TAA would promptly recall the memory T cells able to eliminate or control tumor growth with an improved prognosis.

The relevance of our findings resides in the fact that the microbiota-derived antigens show homology with TAAs shared by several cancers of different histological origin. In particular, MAGE-A3 and MAGE-A10 have been identified in non-small cell lung cancers (NSCLC), bladder cancers, esophageal and head and neck cancers, and sarcomas [34]. In particular, MAGE-A3 is over-expressed in multiple tumor types including melanoma [35] and lung cancer [36] and its presence has been associated with worse prognosis in colorectal cancer [37], cutaneous squamous cell carcinoma [38], and undifferentiated pleomorphic sarcoma/myxofibrosarcoma [39]. Consequently, structural and conformational homology of peptides derived from microbiota species with these TAAs may have a strong impact on several cancers.

These findings open a new horizon in the mutual interaction between the microbiota and immune response in humans with a potential profound impact on tumor development and progression. Moreover, they provide a completely novel class of antigens to be possibly used as anti-cancer preventive vaccination, even administered as food integrators.

If confirmed, this would completely revolutionize the cancer immunotherapy field and represent a milestone in fighting cancer.

## Supplementary Information

**Additional file 1: Figures S1–4.** Percentage (average) of type of residues at each position of the microbiota-derived epitopes when aligned to the corresponding TAA. (A) Identical residues; (B) conservative residues; (C) non-conservative residues; (D) Identical + conservative residues. **Figure S5.** Affinity to HLA-A* 02:01 and 24:02 molecules of each microbiota-derived epitopes with homology to the corresponding TAA. The red dotted line indicates the affinity of the TAA. **Figure S6–17.** Predicted 3D conformation of TAA and microbiota-derived paired peptides. The surface conformation of the paired TAA microbiota-derived peptides is shown. Residues in the microbiota-derived epitopes identical to the TAA sequences are indicated in green color. Green areas = contact points with HLA-A molecule; Magenta areas = contact points with TCR α chain; Light Blue = contact points with TCR β chain. The latter are available only in **Figure S6–S13.** (HLA-A* 02:01 restricted epitopes). **Figures S18.** Percentage of identical values (± 10%) of planare and dehidral angles between of the microbiota-derived epitopes and the corresponding TAA. **Figures S19–S24.** Structural predicted backbone conformation of paired TAA and microbiota-derived epitopes. Green areas = contact points with HLA molecule; Magenta areas = contact points with the TCR α chain; light blue areas = contact points with the TCR β chain. The latter are available only in Additional file 1: **Figure S19–22.** (HLA-A* 02:01 restricted epitopes). Values of the planar as well as dihedral angles between atoms of selected TCR-facing residues are shown in parallel.

## Data Availability

Data and material have been deposited and are publicly available at 10.5281/zenodo.6759952.
