# Supplementary material for: Molecular mimicry between tumor associated antigens and microbiota-derived epitopes
Source: J Transl Med. 2022 Jul 14;20:316. doi: 10.1186/s12967-022-03512-6 (PMC9281086; doi:10.1186/s12967-022-03512-6)

Suppl. Fig. 1

IDENTICAL RESIDUES

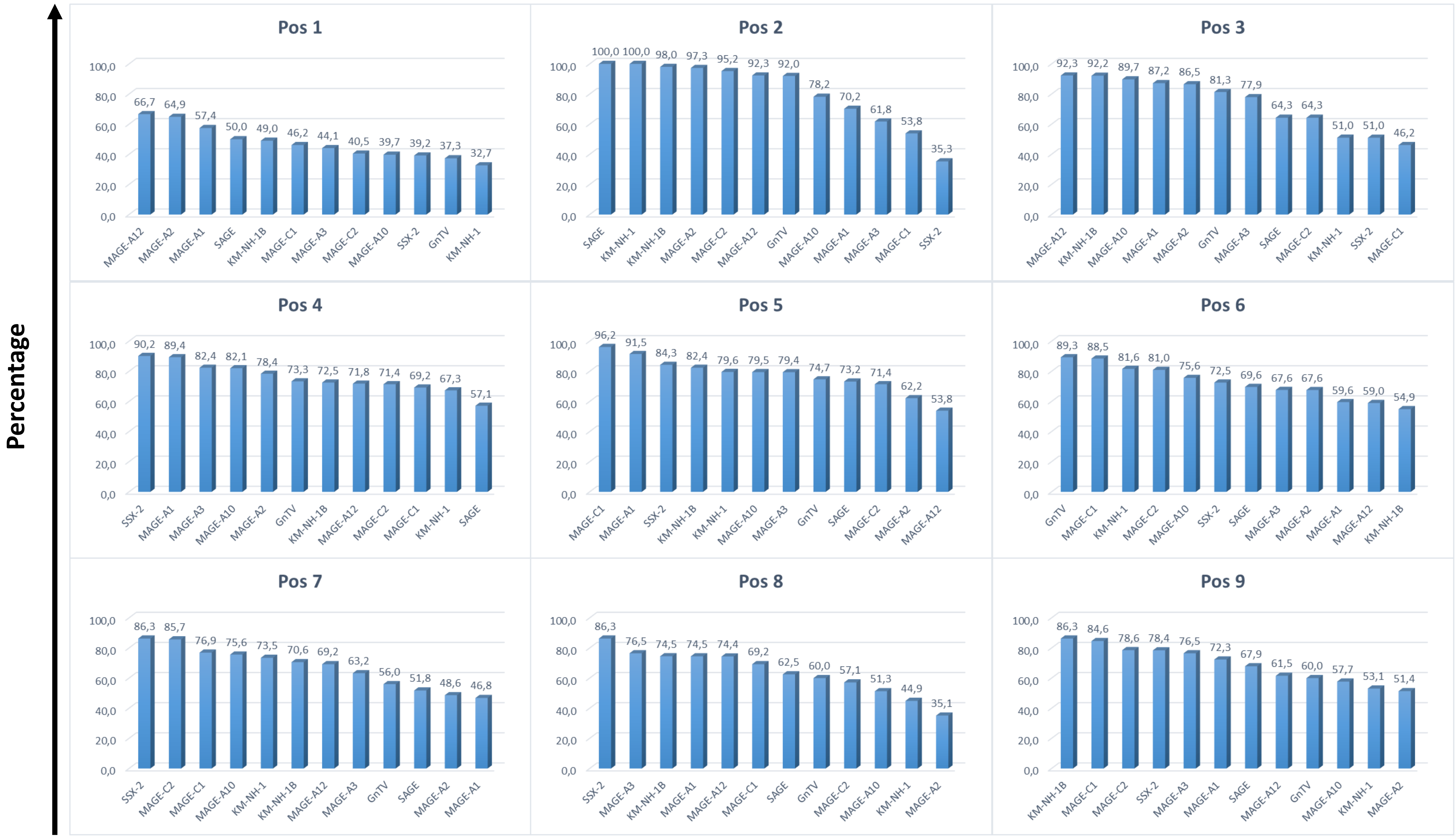

Suppl. Fig. 2

CONSERVATIVE RESIDUES

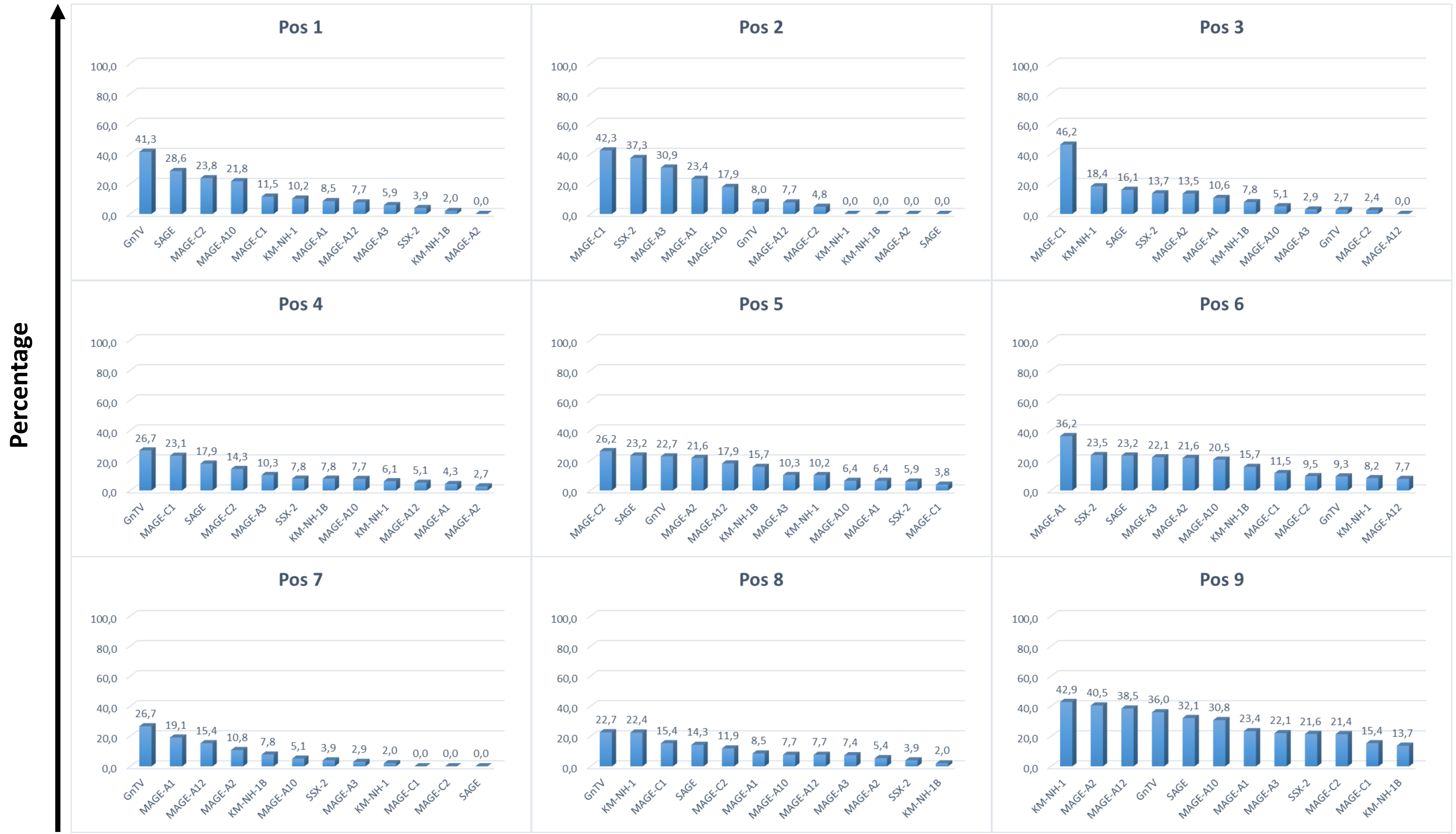

Suppl. Fig. 3

NON-CONSERVATIVE RESIDUES

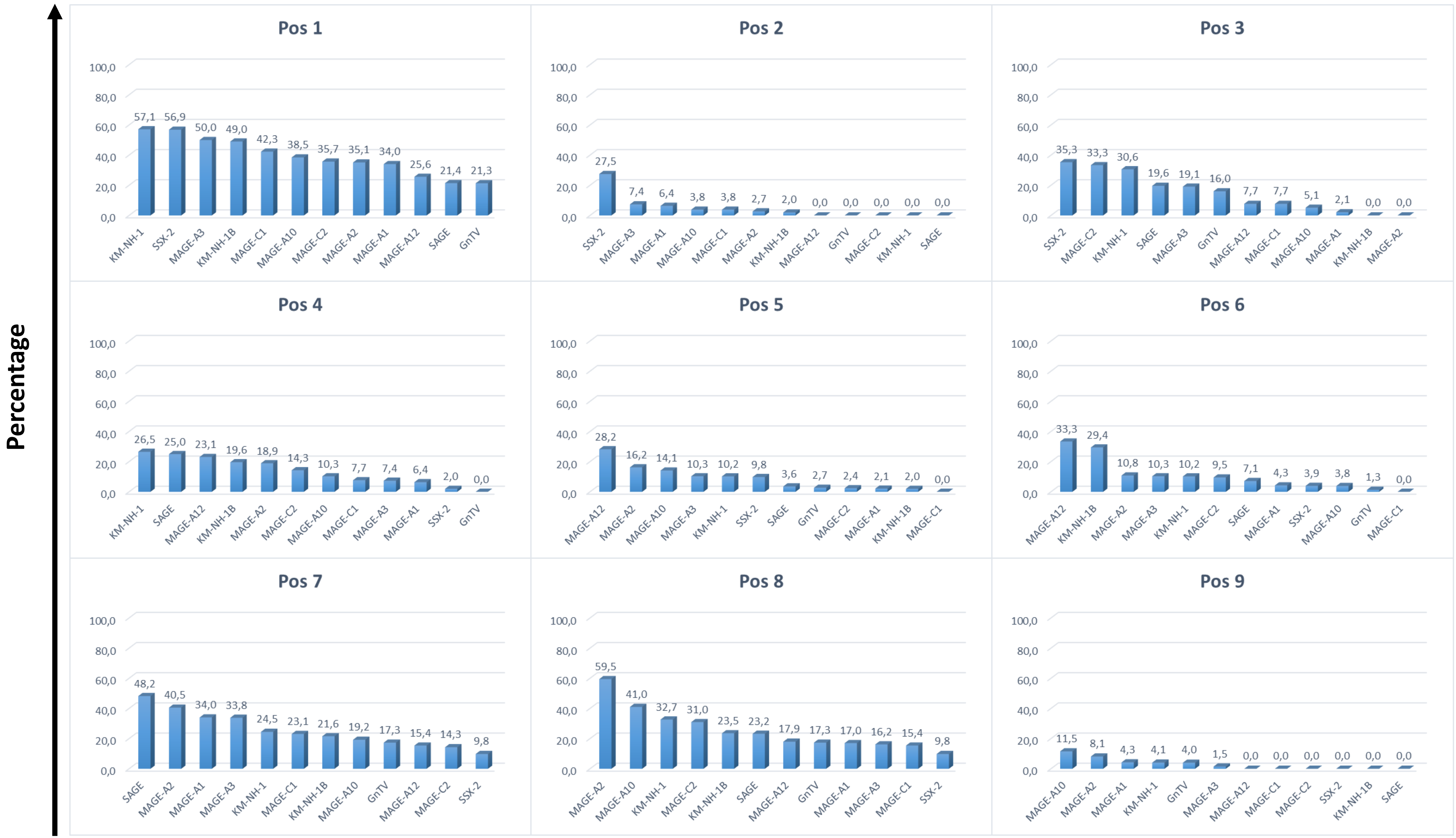

Suppl. Fig. 4

IDENTICAL + CONSERVATIVE RESIDUES

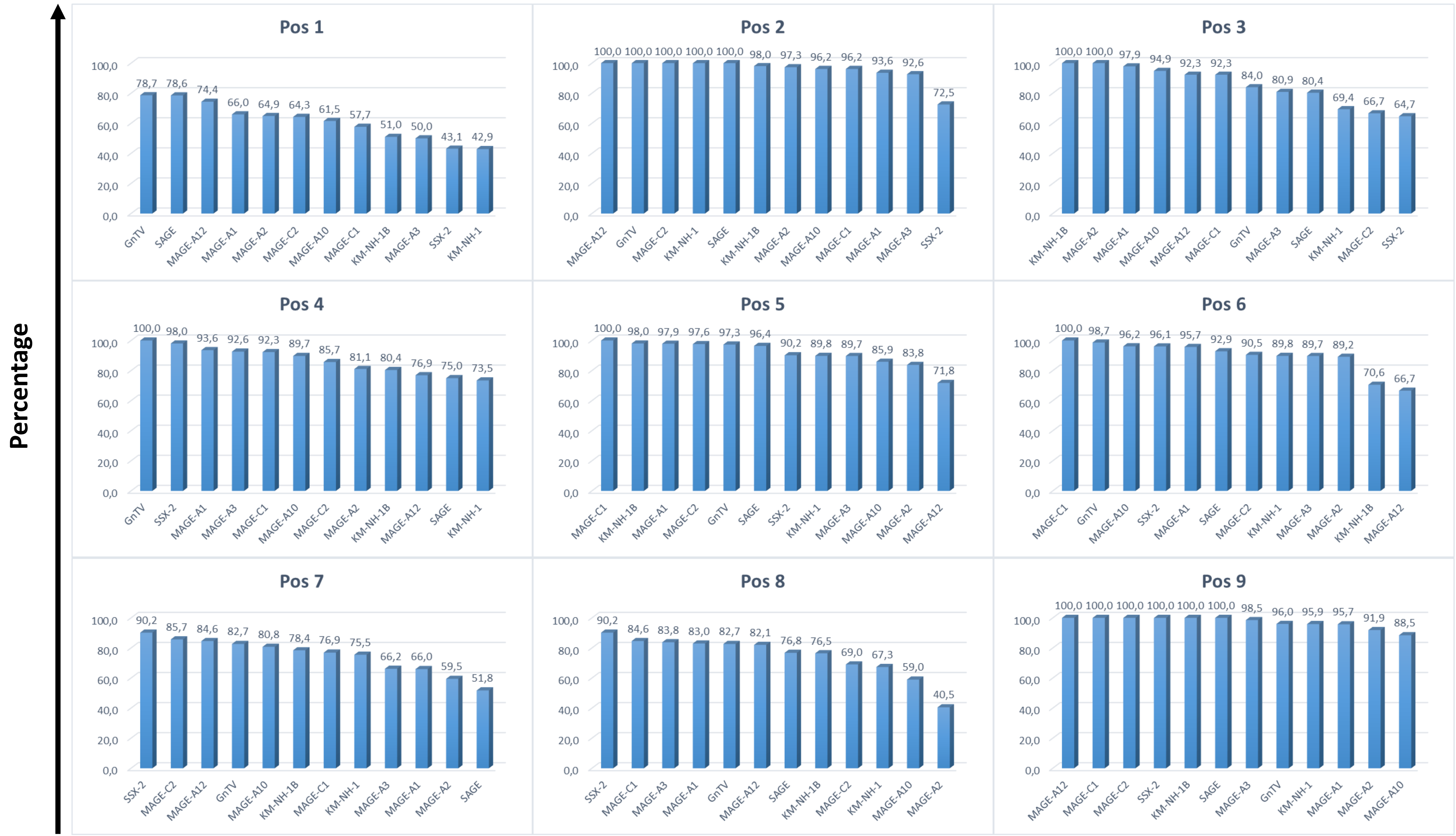

Suppl. Fig. 5

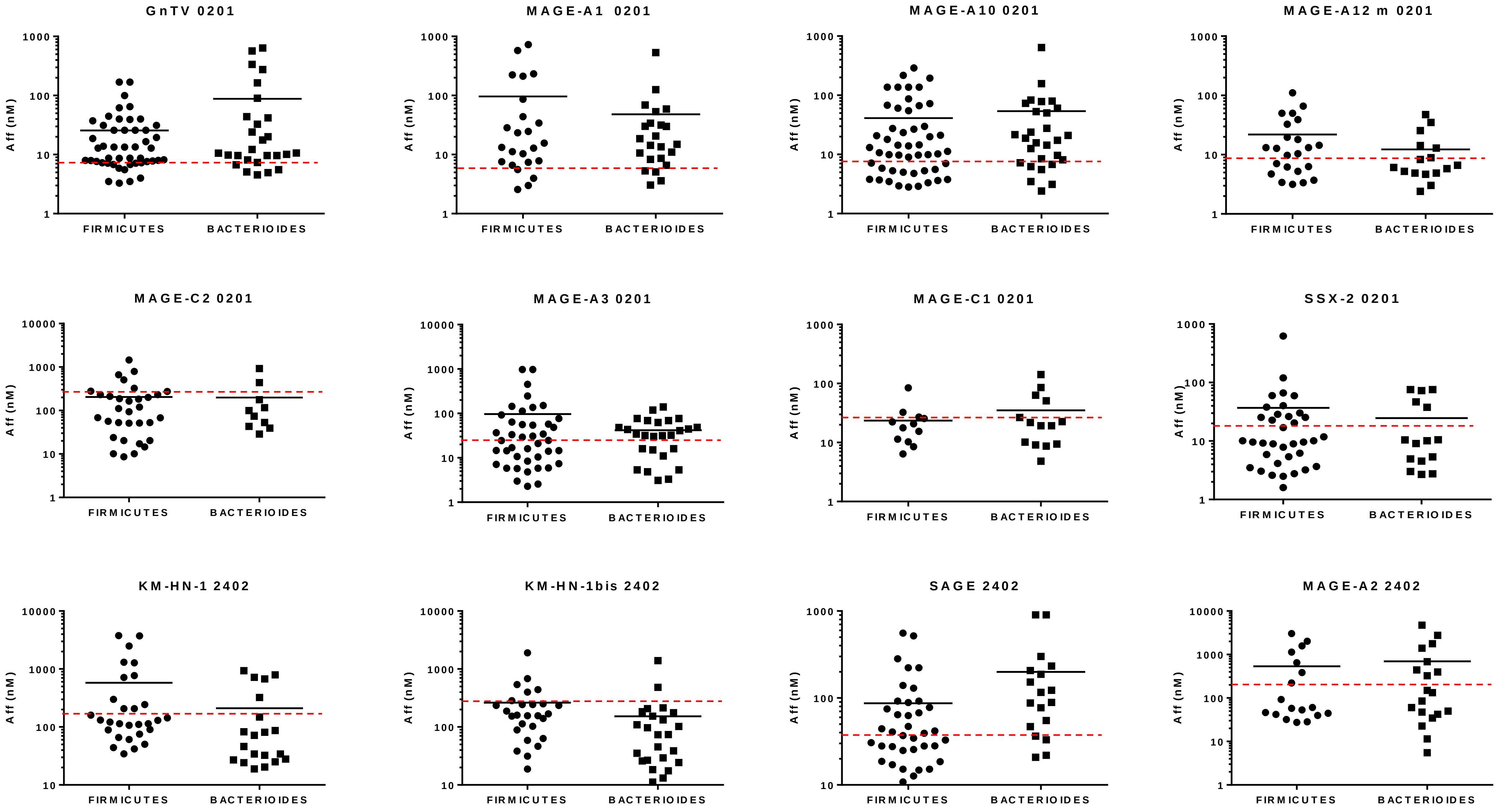
GnTV 0201
MAGE-A1 0201
MAGE-A10 0201
MAGE-A12 m 0201
MAGE-C2 0201
MAGE-A3 0201
MAGE-C1 0201
SSX-2 0201
KM-HN-1 2402
KM-HN-1bis 2402
SAGE 2402
MAGE-A2 2402
Aff (nM)
FIRMICUTES
BACTERIOIDES
1
10
100
1000
10000

Suppl. Fig. 6

MAGE A-10

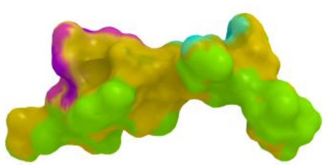

GLYDGMEHL - TAA

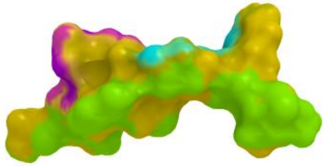

GLYDGMEYI FIRM

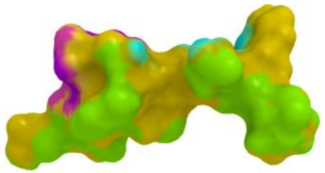

GLYDSMEYV FIRM

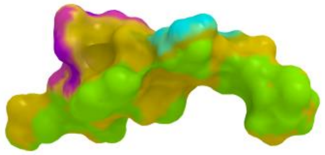

GLYDGMLGV FIRM

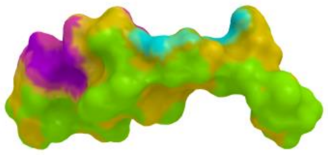

YLYDDMEHL FIRM

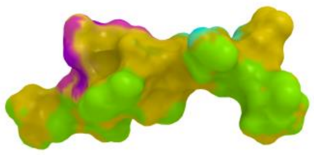

ALYDGMEEV FIRM

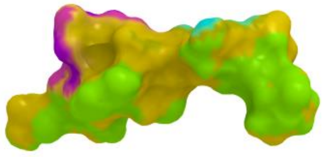

GLYDGMNTV FIRM

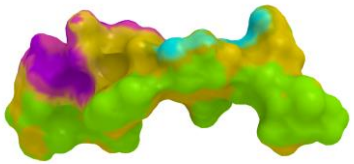

YLYNGIHEI FIRM

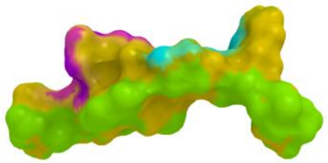

VMYDGMEYL FIRM

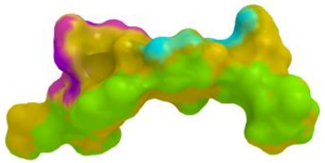

GLYDGVEHV FIRM

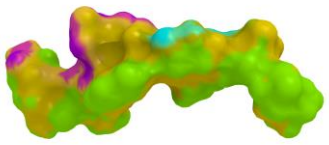

KLYDGLESL FIRM

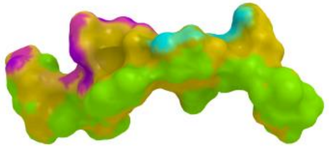

KLYDGIEHL FIRM

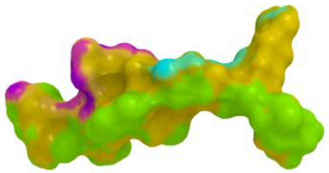

MLYDGMERL FIRM

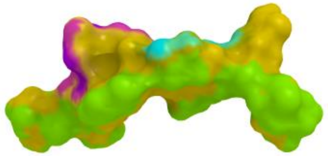

SLYDGLEQL FIRM

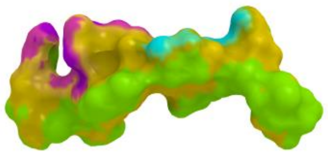

RLYDGLEHL FIRM

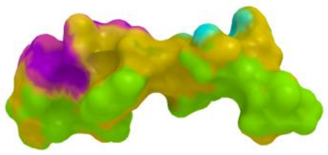

YLYDGLDHI BACT

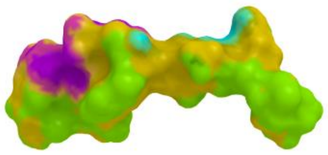

FLYDSVAHL BACT

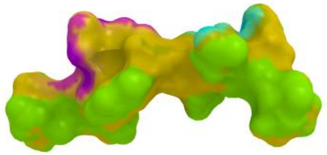

VLYDGVAHL BACT

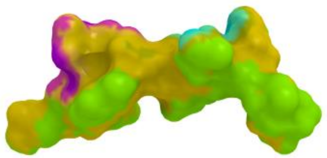

GMYDGLEHV BACT

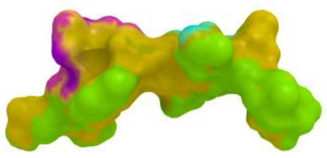

GLYDGLENL BACT

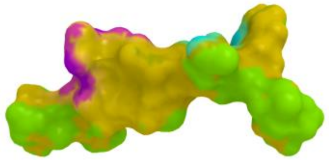

TLADGMEYL BACT

Suppl. Fig. 7

MAGE A-3

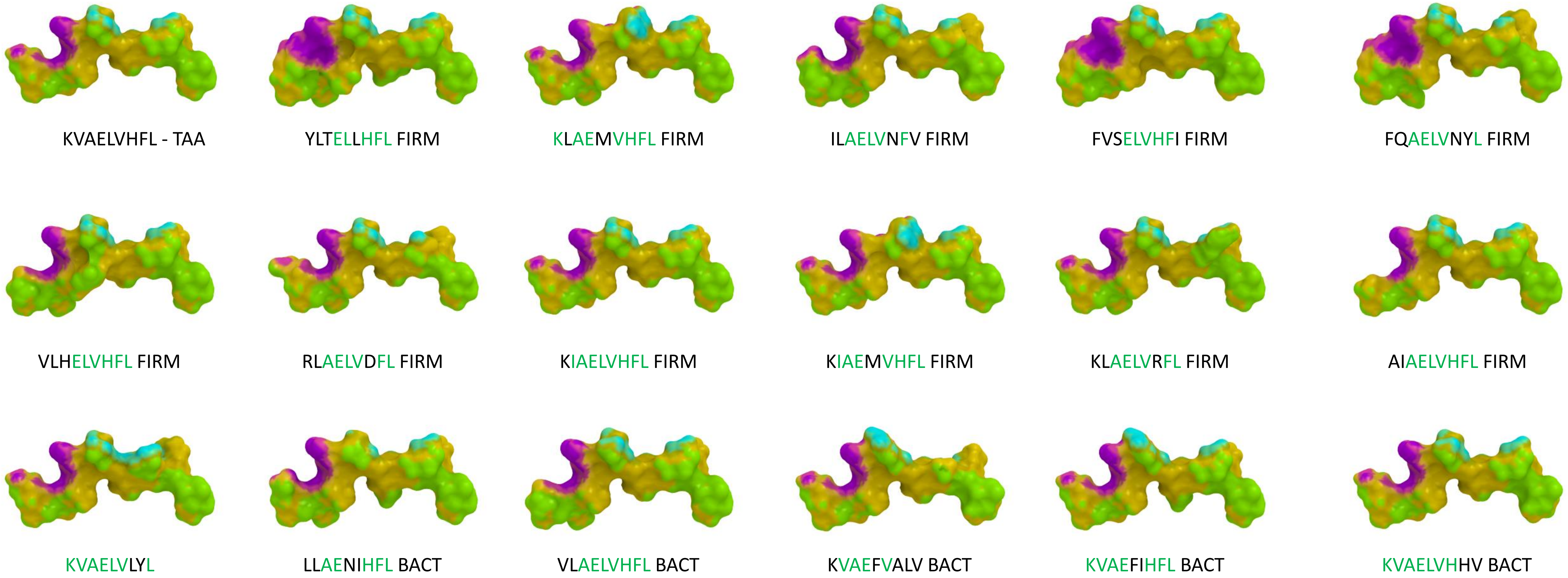

Suppl. Fig. 8

MAGE A-12

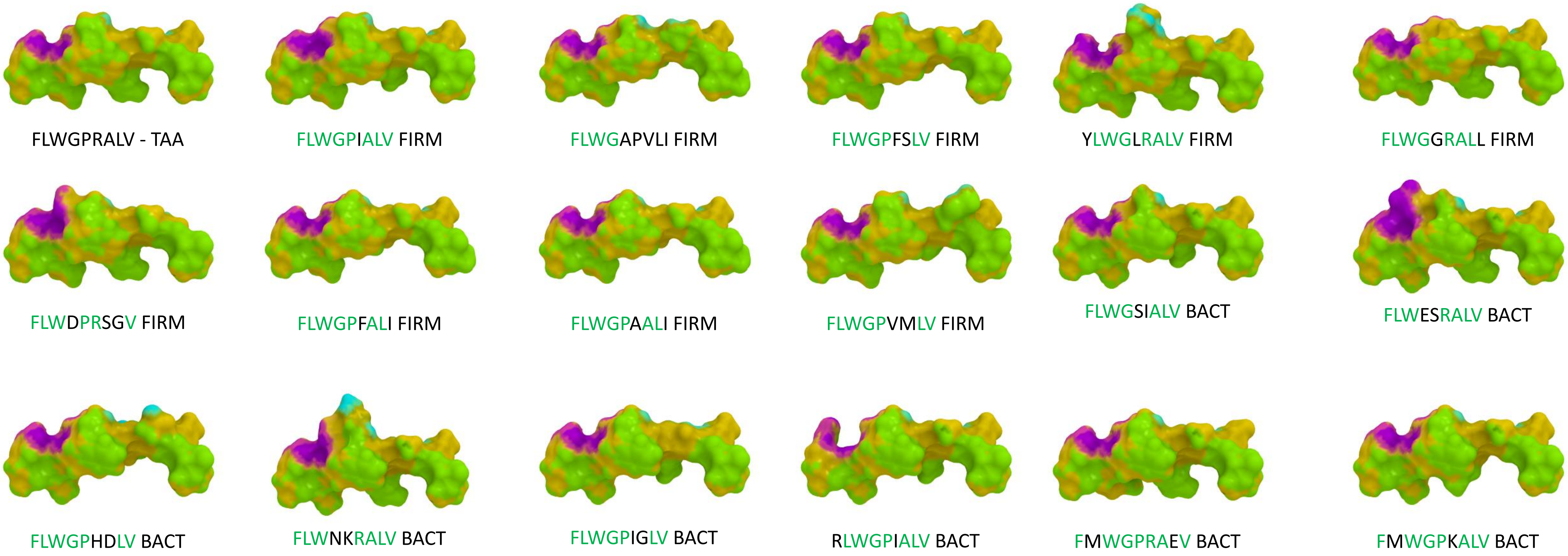

Suppl. Fig. 9

MAGE A-1

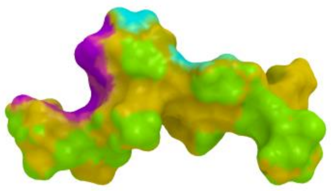
MAGE A 1- TAA

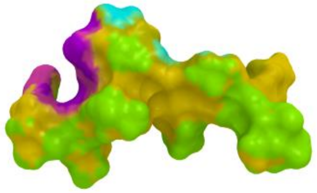
RLLEYIVKI FIRM

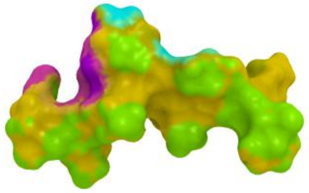
RLLDLIKV FIRM

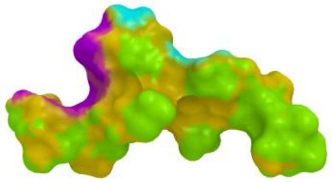
KVMEYLISI FIRM

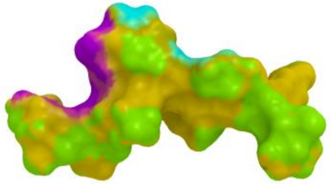
KVLEYVIRV FIRM

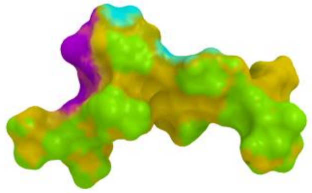
ALLEYVIKV BACT

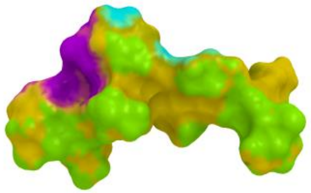
HLLEYVIKV BACT

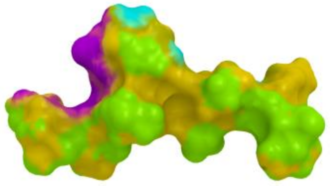
KVLEYLPKV BACT

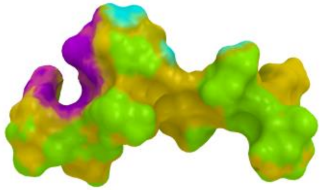
RILEYVIKV BACT

Suppl. Fig. 10

SSX-2

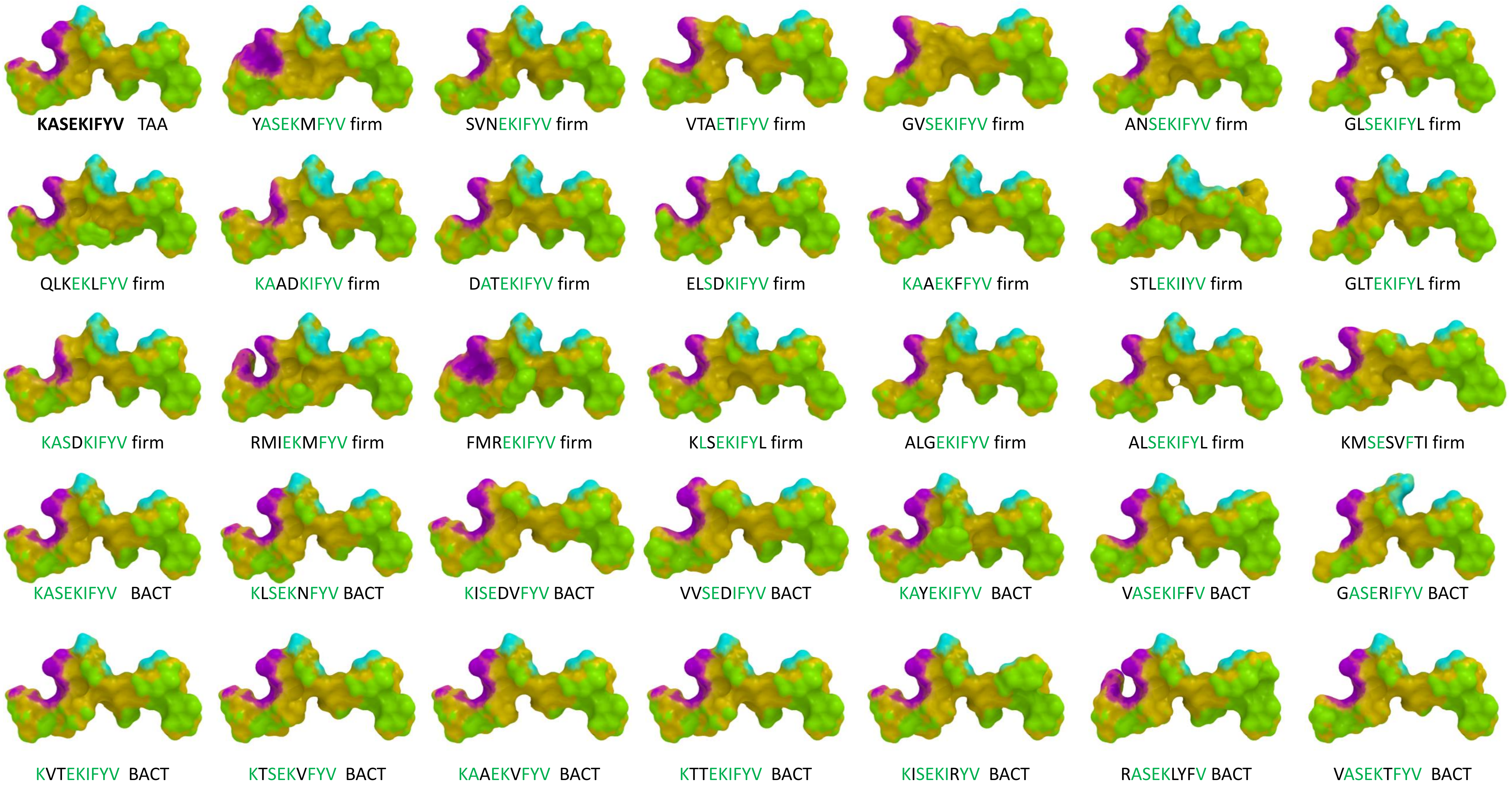

Suppl. Fig. 11

MAGE C-2

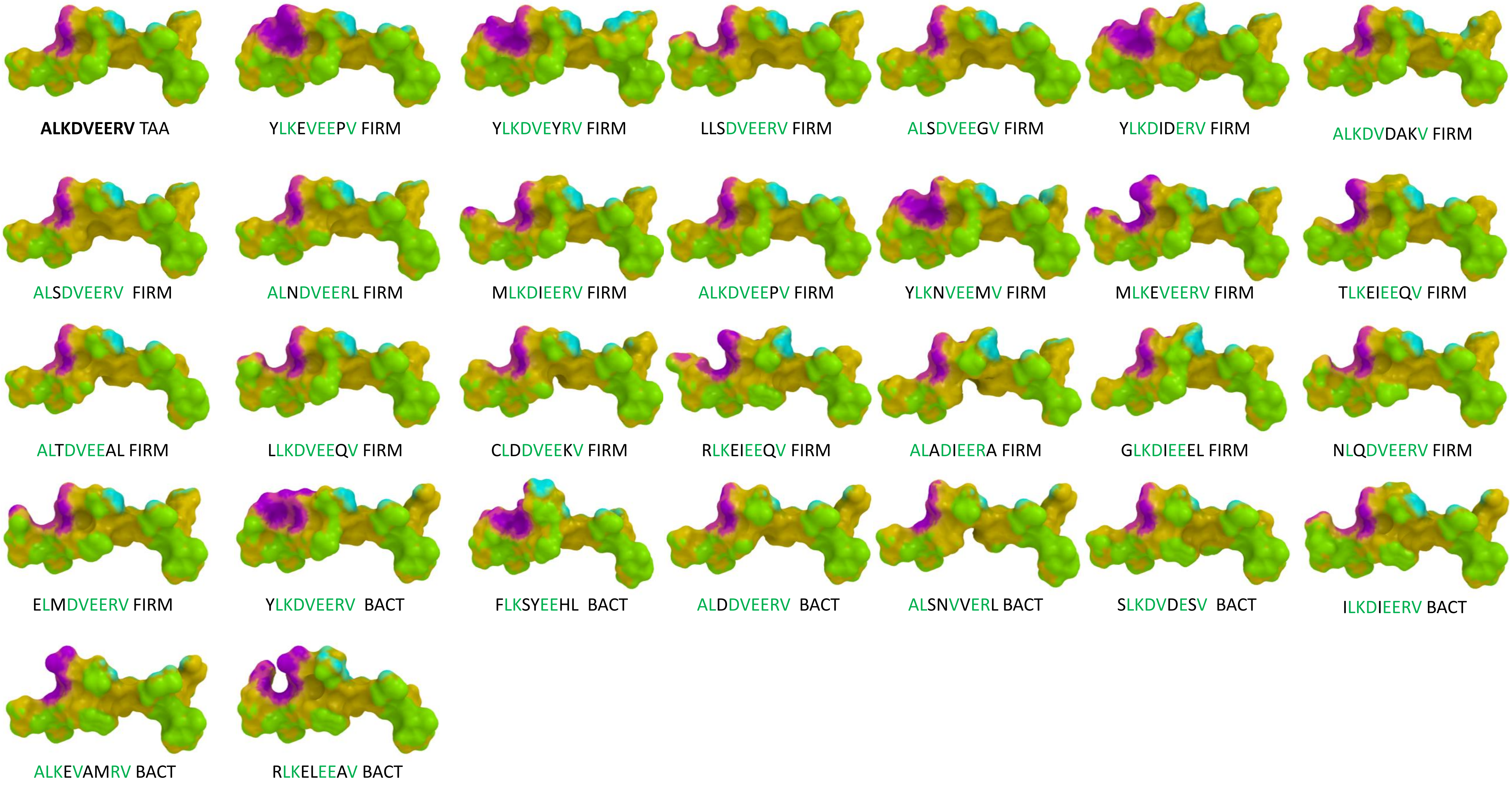

Suppl. Fig. 12

MAGE C-1

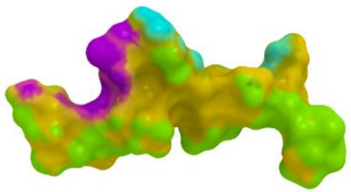

KVVEFLAML TAA

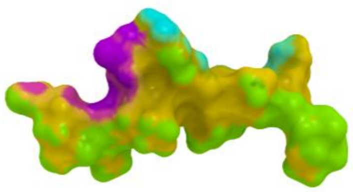

RLVEFLAML FIRM

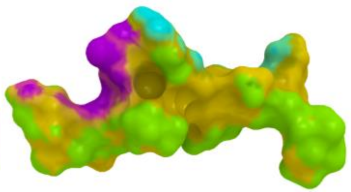

KILEFLAML FIRM

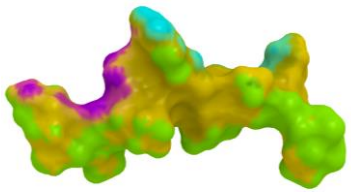

KLVAFLAML FIRM

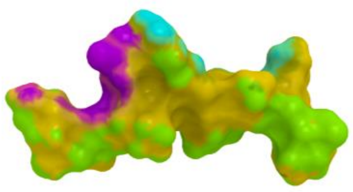

KTVEFLAMV FIRM

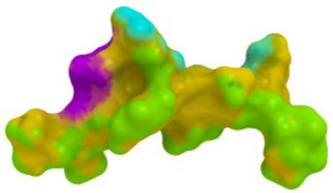

ALFTFLAML FIRM

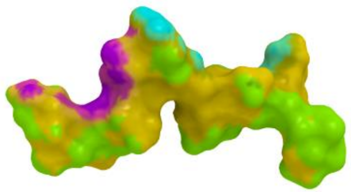

KVADFLAML FIRM

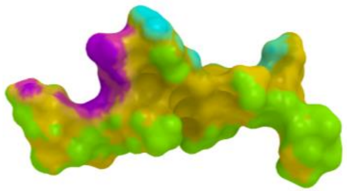

KVIEFLSML FIRM

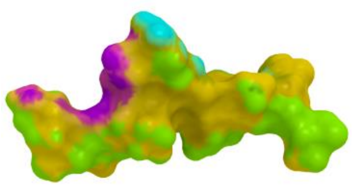

KIVDFLAQA FIRM

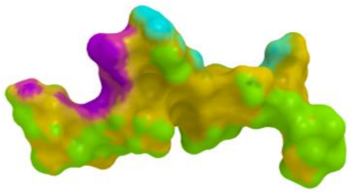

KVVEFLAML FIRM

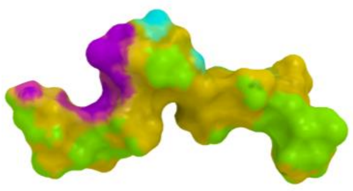

KVAEFVALV BACT

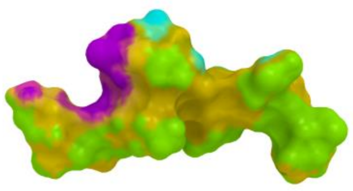

KVIEFLQDV BACT

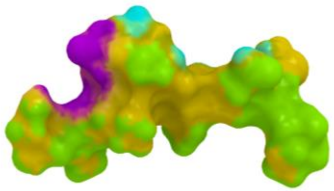

NLVEFIEML BACT

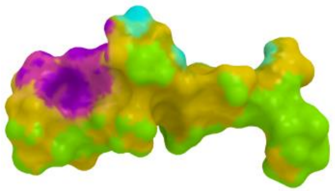

RIVDFLAQL BACT

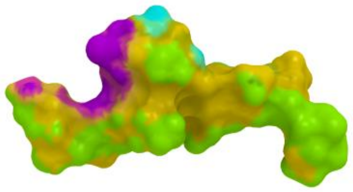

KVIEFLALL BACT

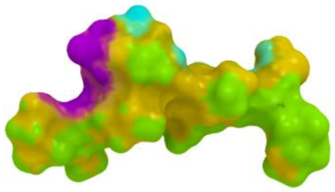

CLNEFLPML BACT

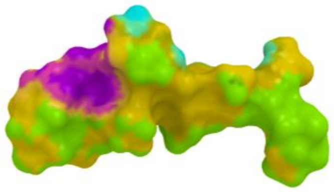

FVVDFLAQL BACT

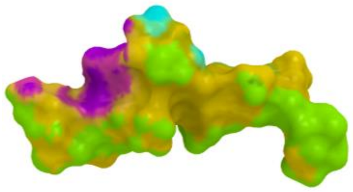

KVVDFLALL BACT

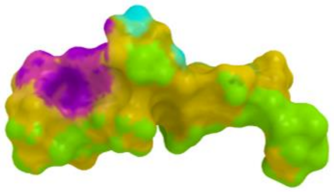

RIVDFLALL BACT

Suppl. Fig. 13

GnTv

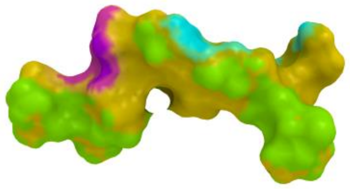

VLPDVFIRV TAA

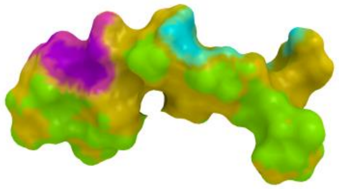

YLPDIFIRV FIRM

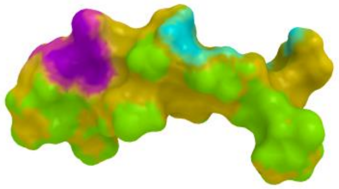

FLQEIFIRV FIRM

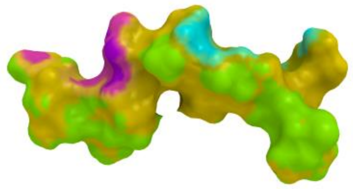

RLPDIFIRV FIRM

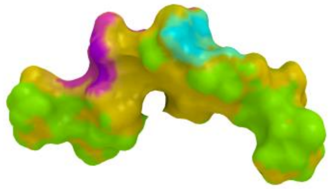

VLPDMFISV FIRM

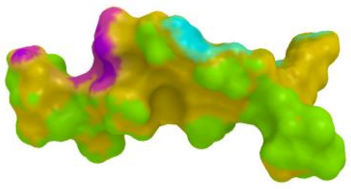

KLVDVLIRI FIRM

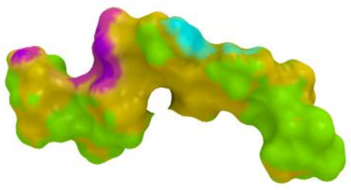

KLPDVFVSI FIRM

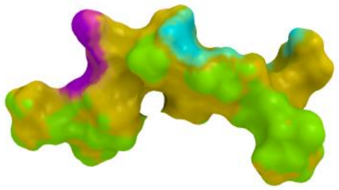

ALPEIFIRV FIRM

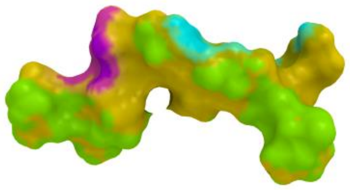

VLPDVFIRV FIRM

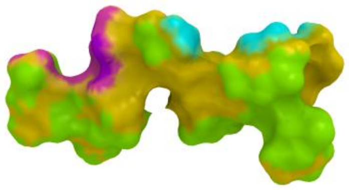

LLPDVLFRV FIRM

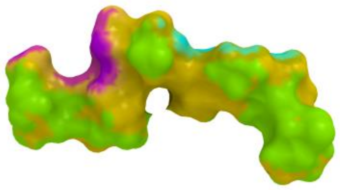

IMPDVFVPV BACT

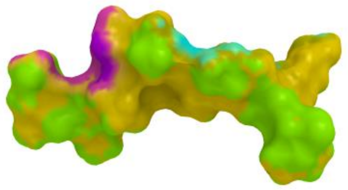

LLSDVFIRV BACT

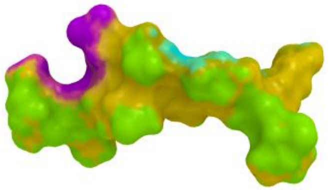

ILQEVFIRV BACT

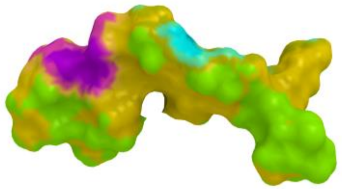

FLPDIFIRI BACT

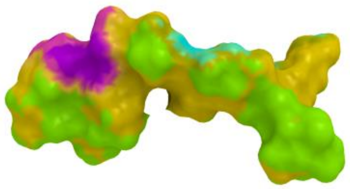

FLPDVFIRI BACT

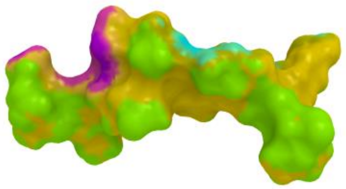

ILQDVFIRV BACT

Suppl. Fig. 14

KM-NH-1

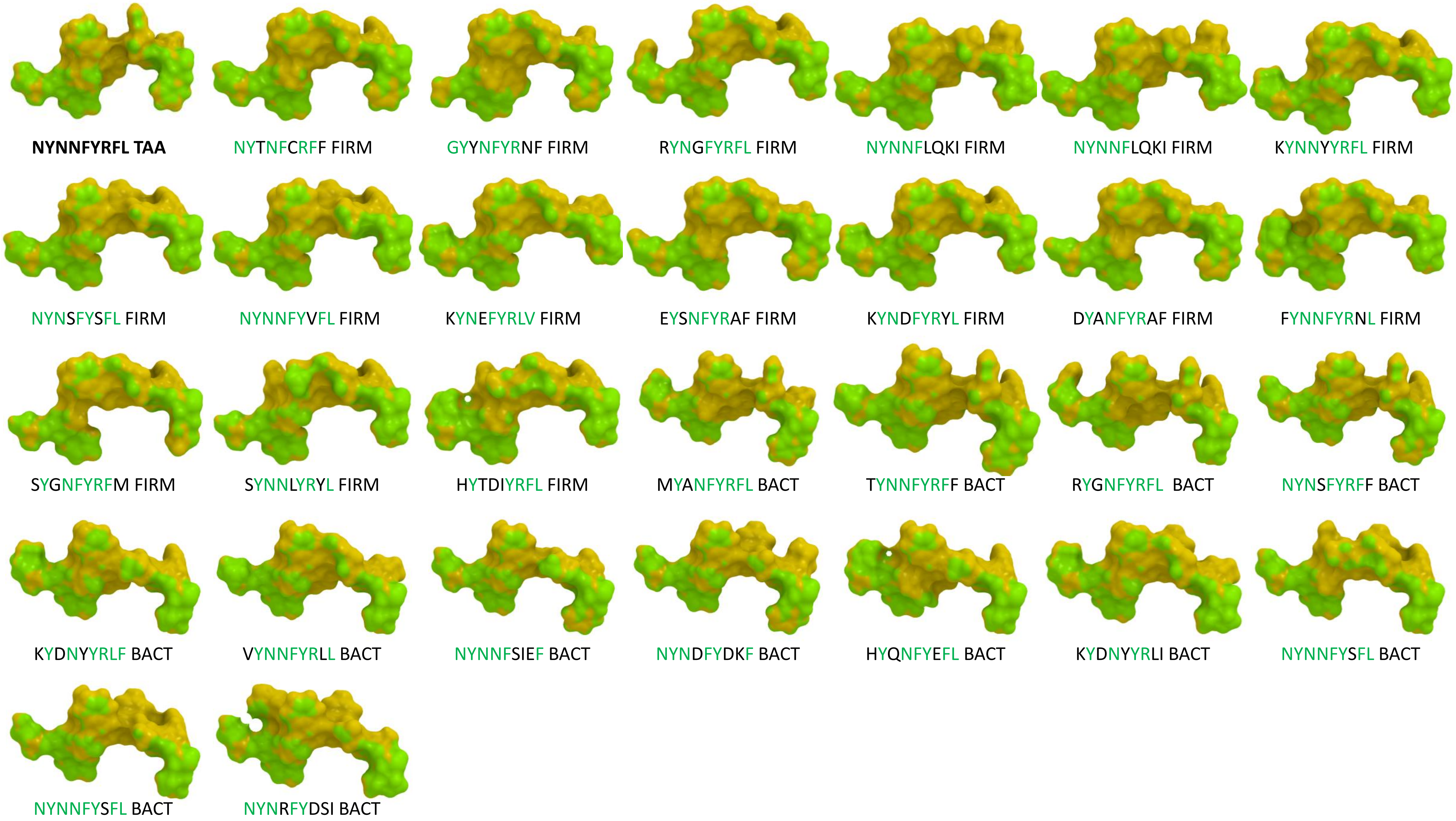

Suppl. Fig. 15

KM-HN1B

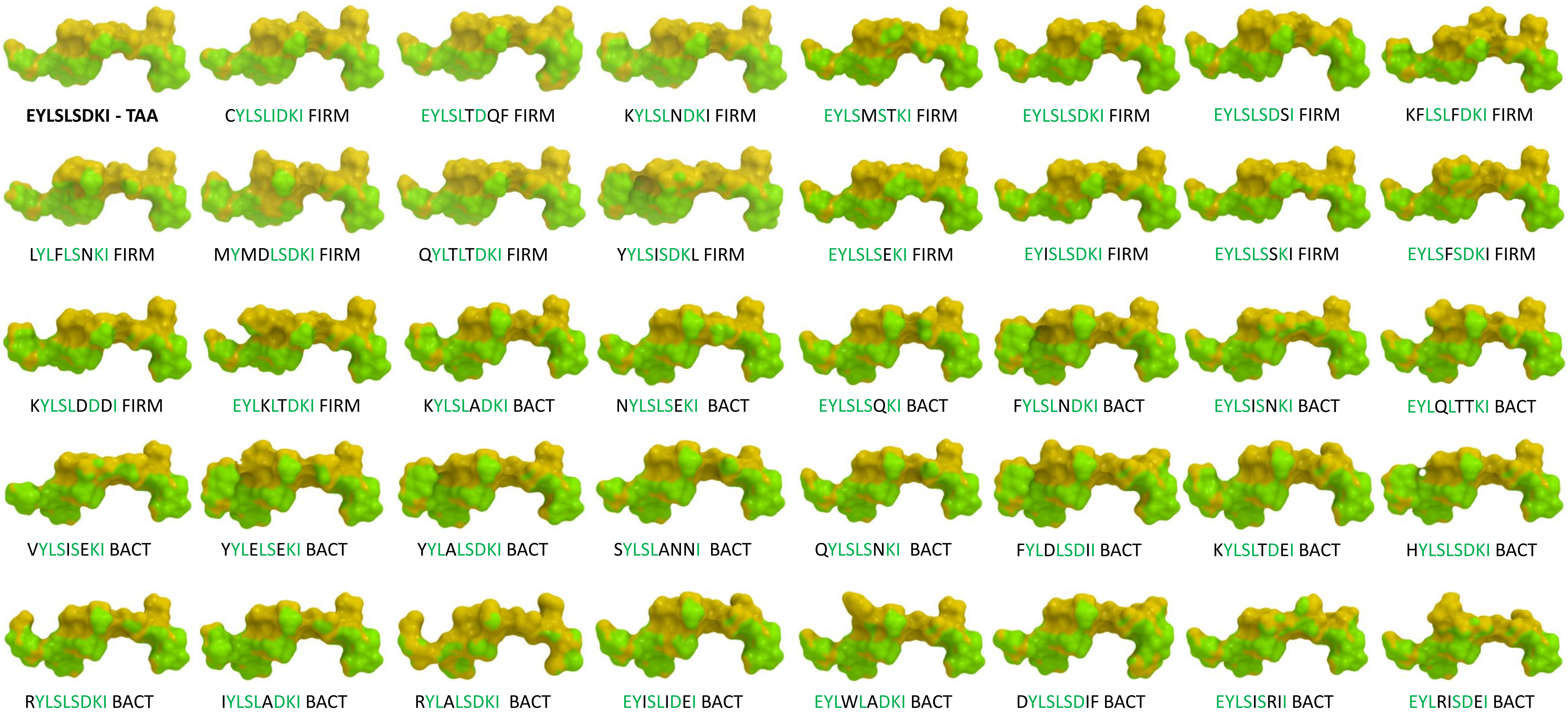

Suppl. Fig. 16

MAGE- A2

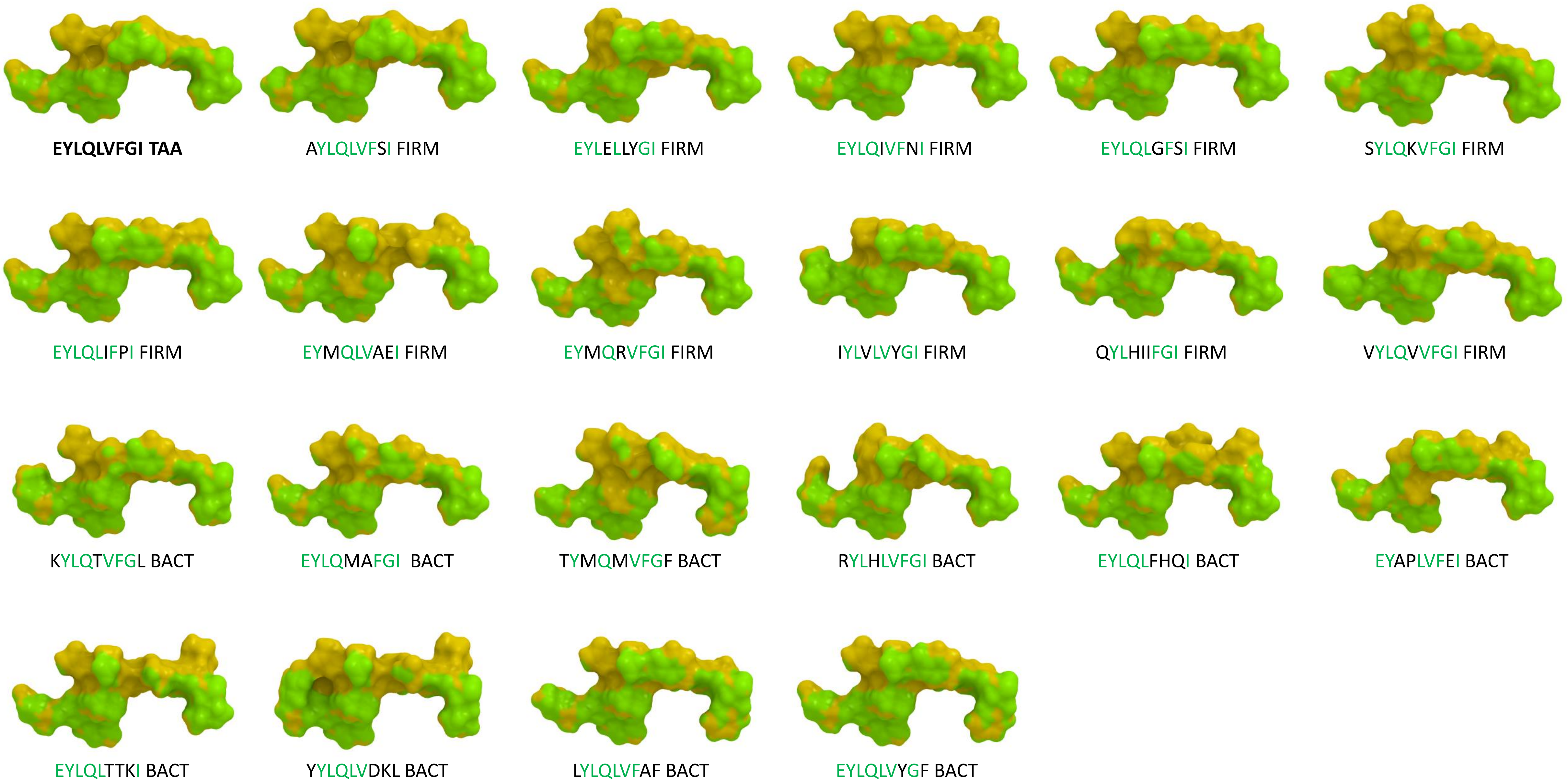

Suppl. Fig. 17

SAGE

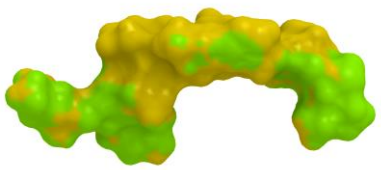

LYATVIHDI TAA

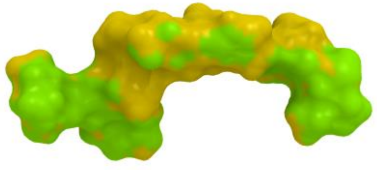

IYAAIIHDI FIRM

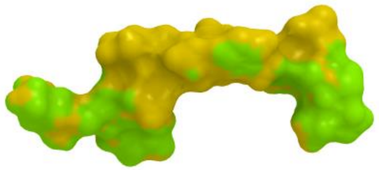

LYATAIHEI FIRM

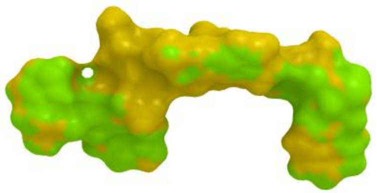

HYANVIHSW FIRM

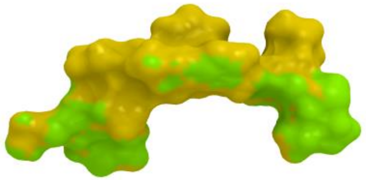

GYATVFHKI FIRM

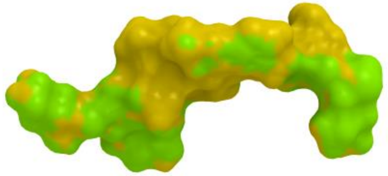

LYAAVLHEL FIRM

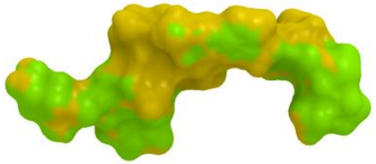

LYASVLHDI FIRM

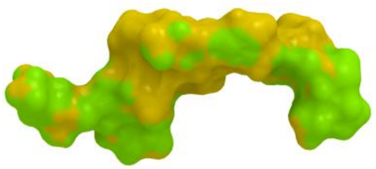

LYAAILHDI FIRM

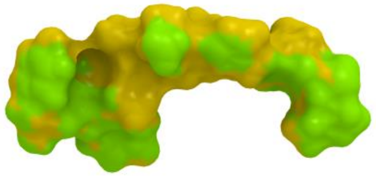

YYASLIHDI FIRM

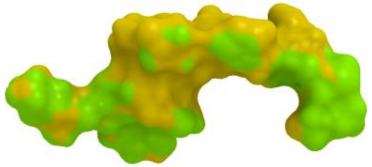

LYQSEIHHI FIRM

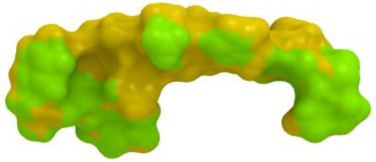

YYATLIHDI FIRM

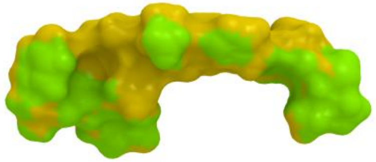

YYATLVHDI FIRM

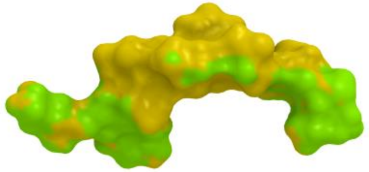

LYATVFQDI FIRM

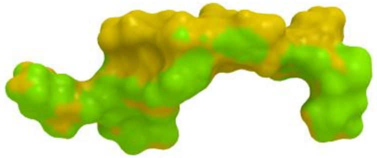

LYFTVIHLL FIRM

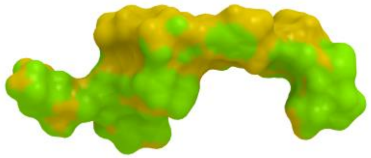

LYLTIIHDI FIRM

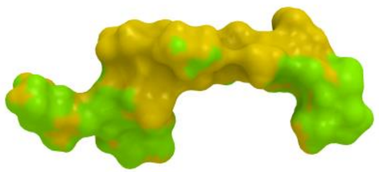

LYATVIAEI FIRM

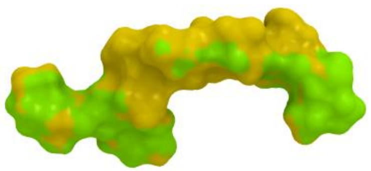

KYAAVIEEI BACT

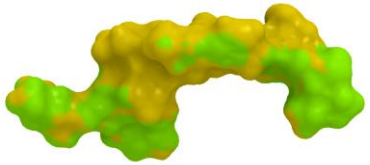

LYSTVIHEI BACT

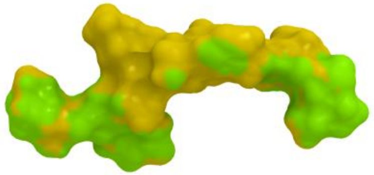

MYALSIHDI BACT

Suppl. Fig. 18

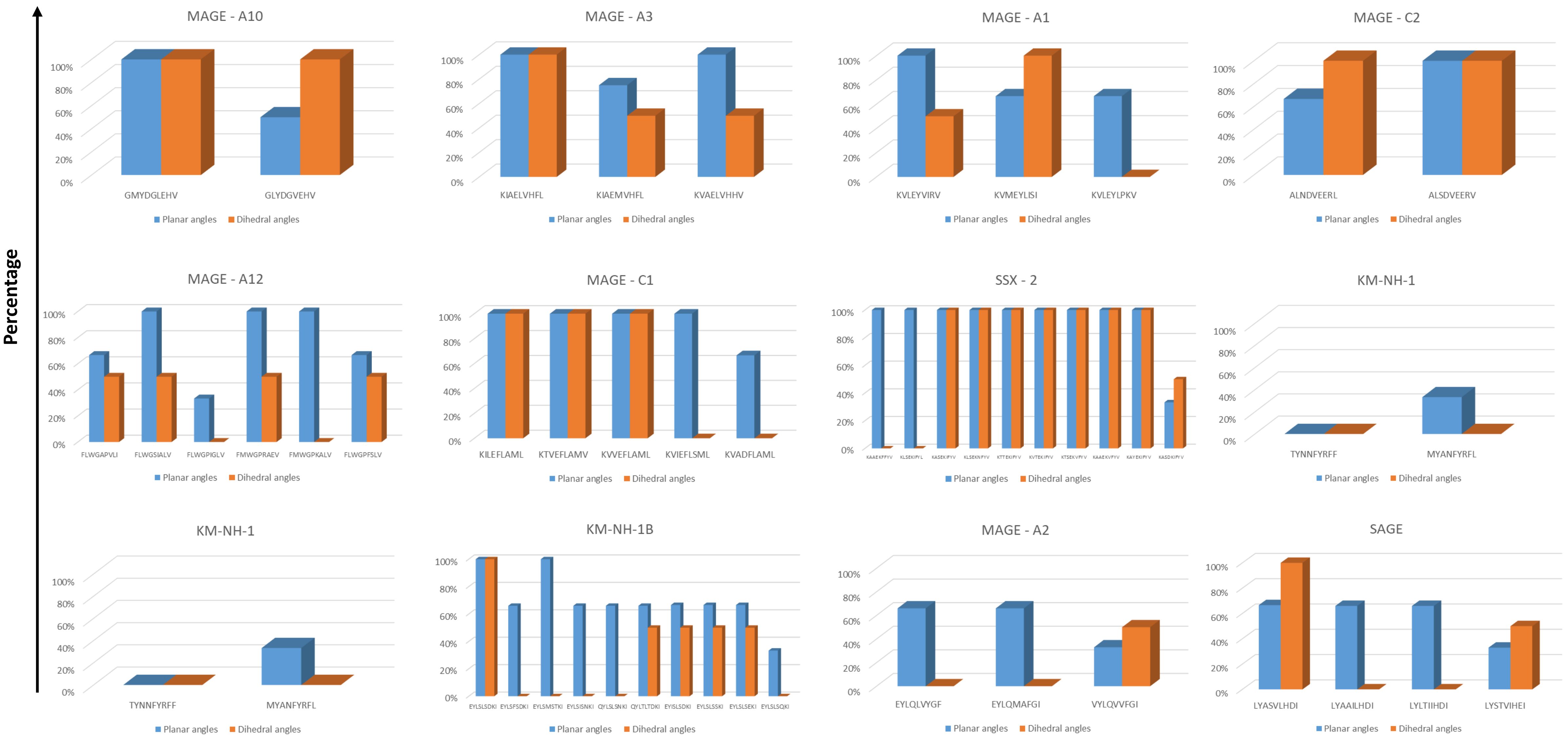
Percentage
MAGE - A10
GMYDGLEHV
GLYDGVEHV
MAGE - A3
KIAELVHFL
KIAEMVHFL
KVAELVHHV
MAGE - A1
KVLEYVIRV
KVMEYLISI
KVLEYLPKV
MAGE - C2
ALNDVEERL
ALSDVEERV
MAGE - A12
FLWGAPVLI
FLWGSIALV
FLWGPIGLV
FMWGPRAEV
FMWGPKALV
FLWGPFSLV
MAGE - C1
KILEFLAML
KTVEFLAMV
KVVEFLAML
KVIEFLSML
KVADFLAML
SSX - 2
KAAEKFFYV
KLSEKIFYL
KASEKIFYV
KLSEKNFYV
KTTEKIFYV
KVTEKIFYV
KTSEKVFYV
KAAEKVFYV
KAYEKIFYV
KASDKIFYV
KM-NH-1
TYNNFYRFF
MYANFYRFL
KM-NH-1
TYNNFYRFF
MYANFYRFL
KM-NH-1B
EYLSLSDKI
EYLSFSDKI
EYLSMSTKI
EYLSISNKI
QYLSLSNKI
QYLTLTDKI
EYISLSDKI
EYLSLSSKI
EYLSLSEKI
EYLSLSQKI
MAGE - A2
EYLQLVYGF
EYLQMAFGI
VYLQVVFGI
SAGE
LYASVLHDI
LYAAILHDI
LYLTIIHDI
LYSTVIHEI
0%
20%
40%
60%
80%
100%
Planar angles
Dihedral angles

Suppl. Fig. 19

MAGE A-10

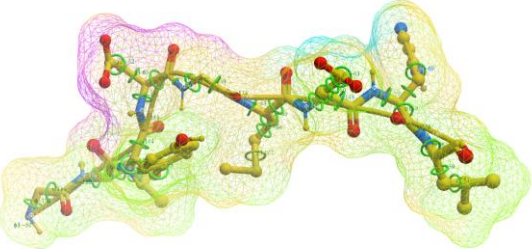

GLYDGMEHL

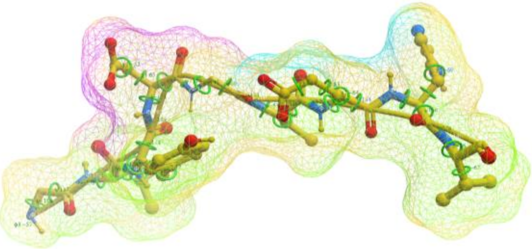

GLYDGVEHV - F

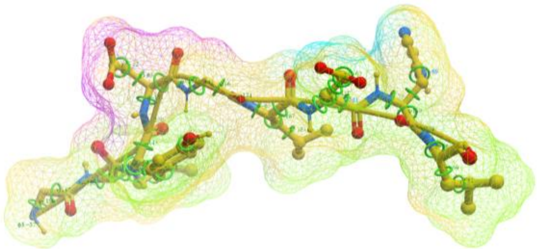

GMYDGLEHV - B

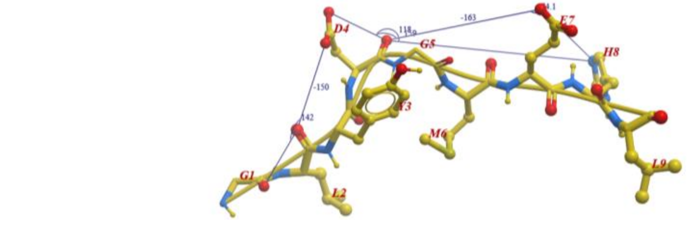

GLYDGMEHL

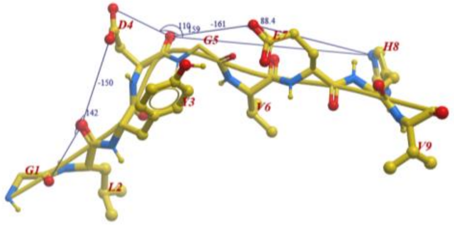

GLYDGVEHV - F

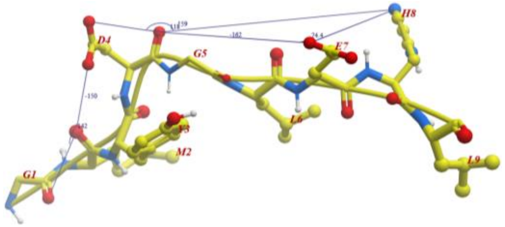

GMYDGLEHL - B

MAGE A-3

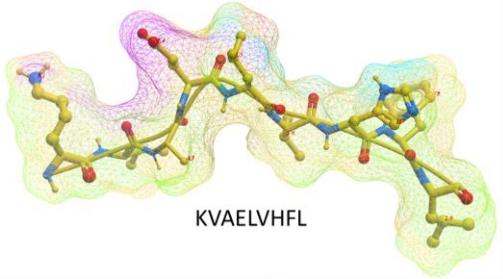

KVAELVHFL

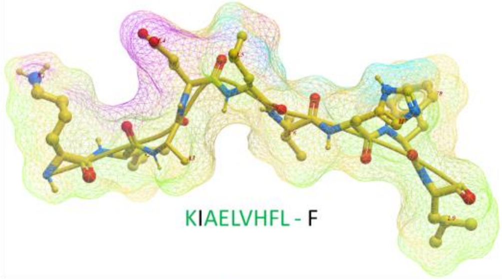

KIAELVHFL - F

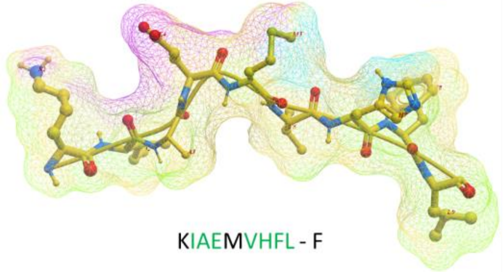

KIAEMVHFL - F

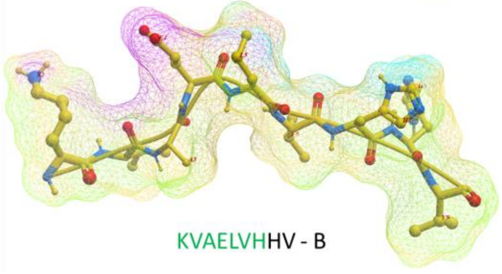

KVAELVHHV - B

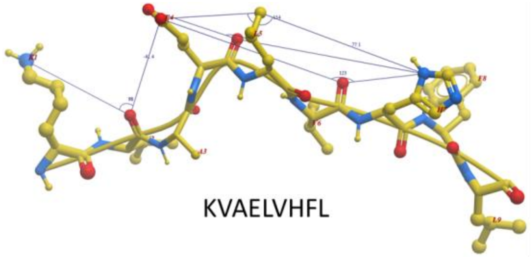

KVAELVHFL

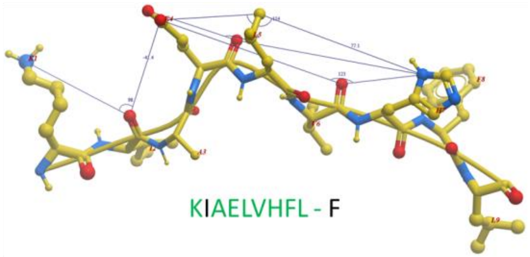

KIAELVHFL - F

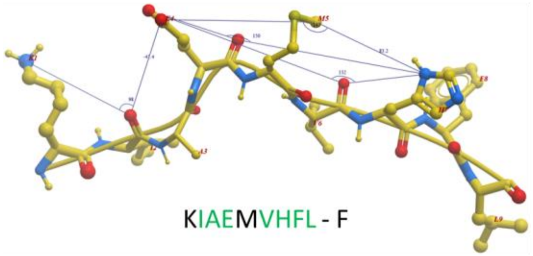

KIAEMVHFL - F

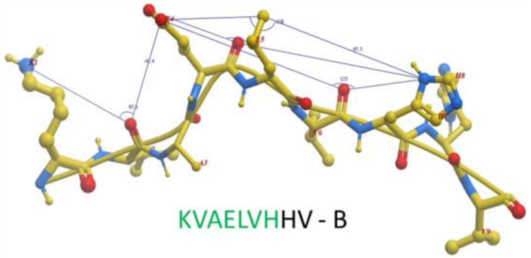

KVAELVHHV - B

Suppl. Fig. 20

MAGE A-1

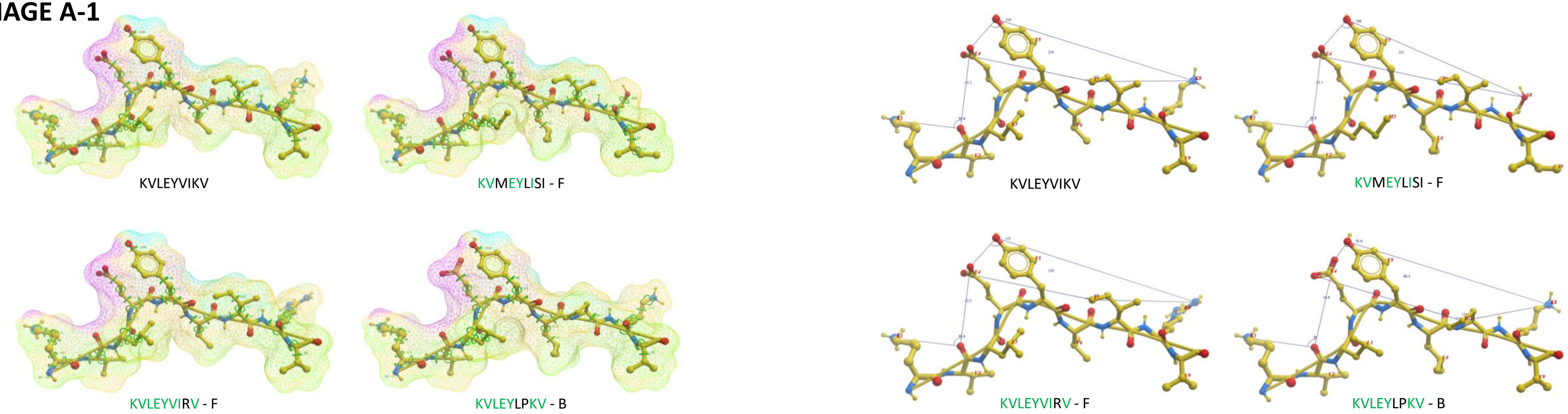

MAGE-C2

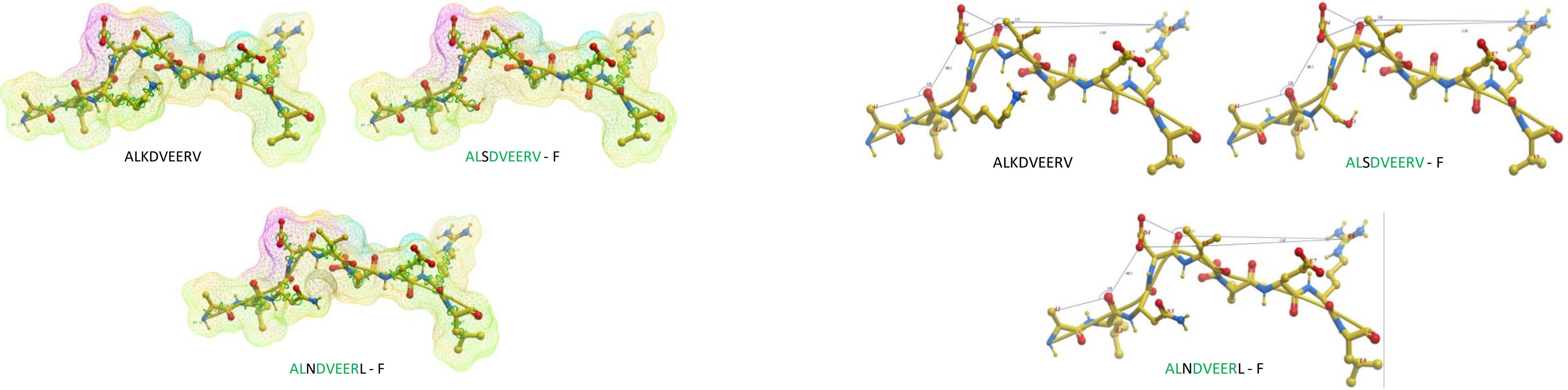

Suppl. Fig. 21

MAGE-A12

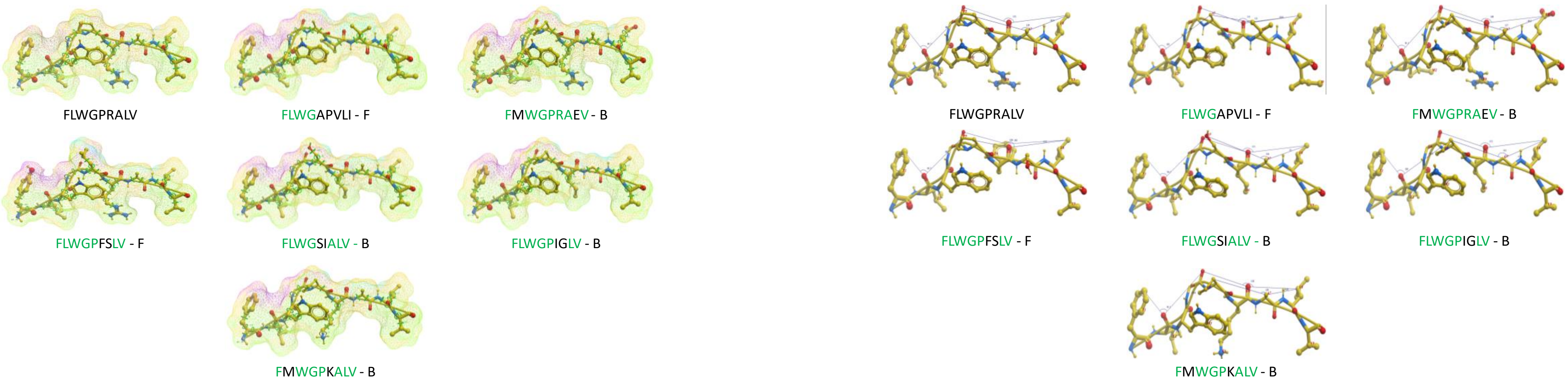

SSX-2

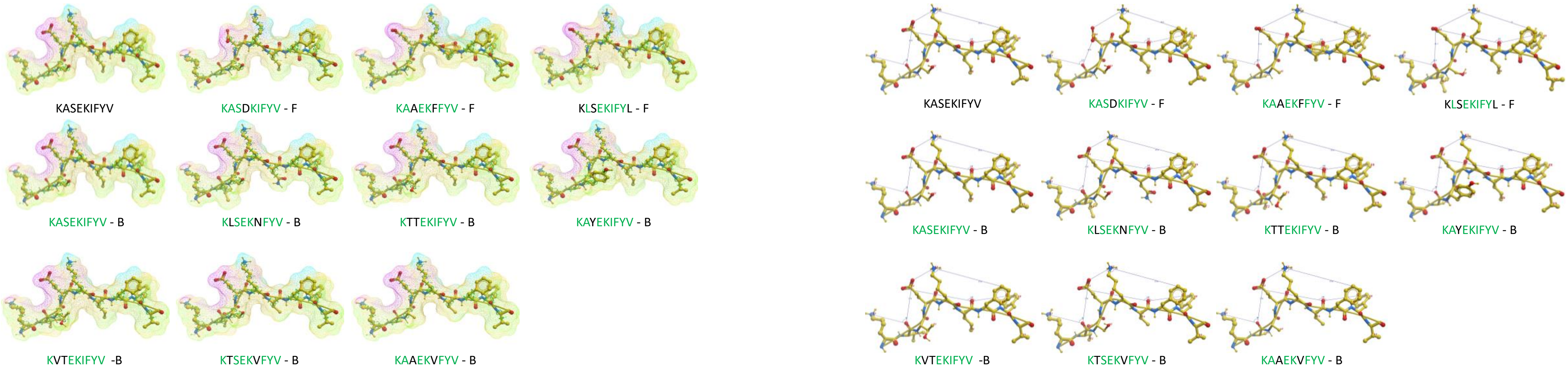

Suppl. Fig. 22

MAGE-C1

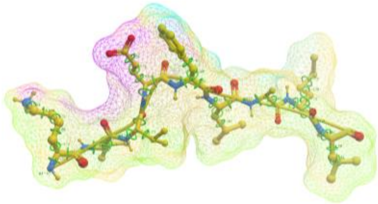

KVVEFLAML

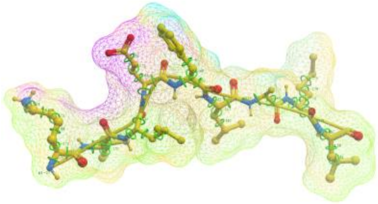

KILEFLAML - F

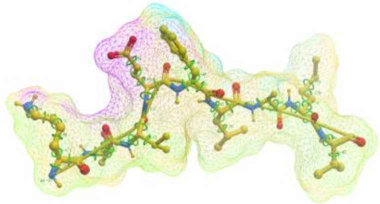

KTVEFLAMV - F

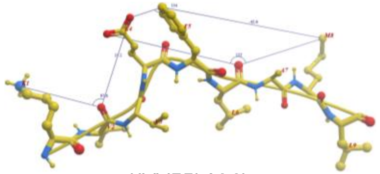

KVVEFLAML

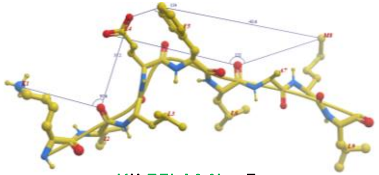

KILEFLAML - F

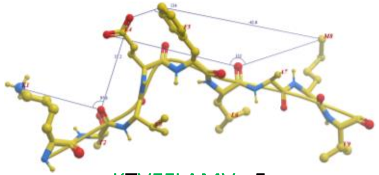

KTVEFLAMV - F

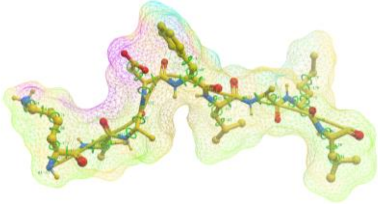

KVADFLAML - F

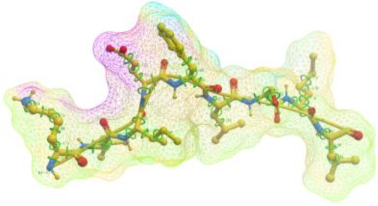

KVIEFLSML - F

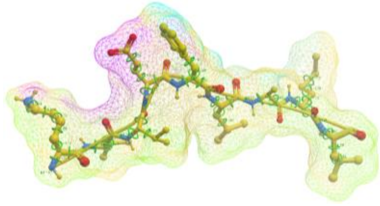

KVVEFLAML - F

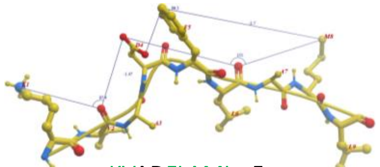

KVADFLAML - F

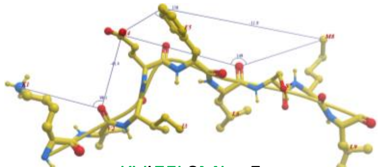

KVIEFLSML - F

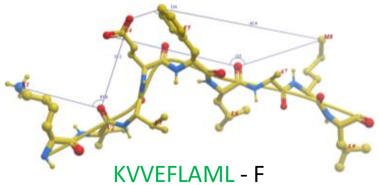

KVVEFLAML - F

Suppl. Fig. 23

KM-HN-1

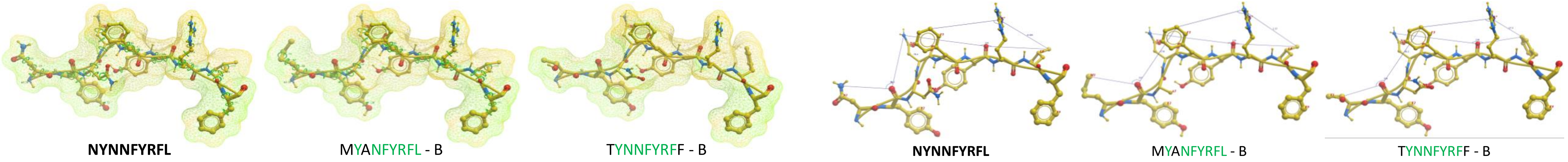

KM-HN-1b

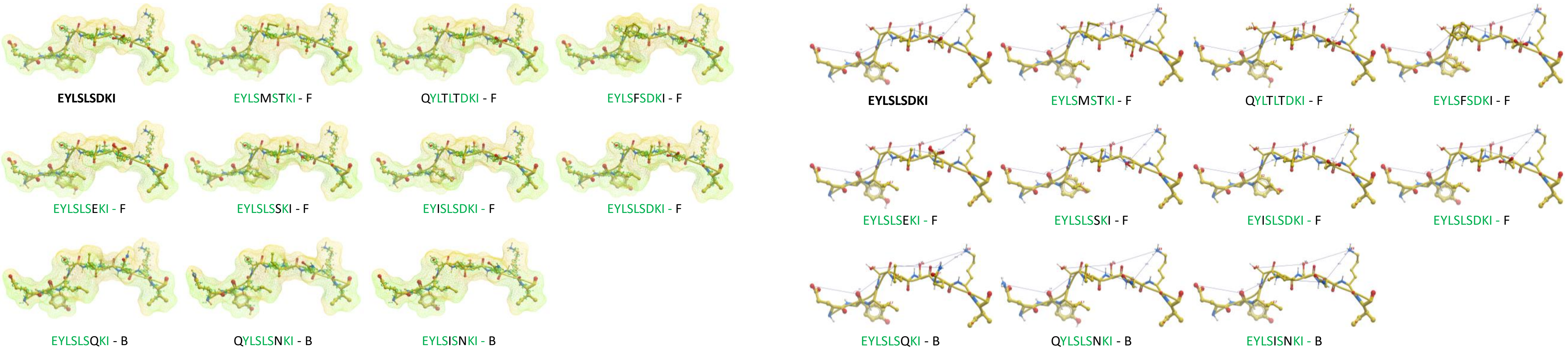

Suppl. Fig. 24

MAGE-A2

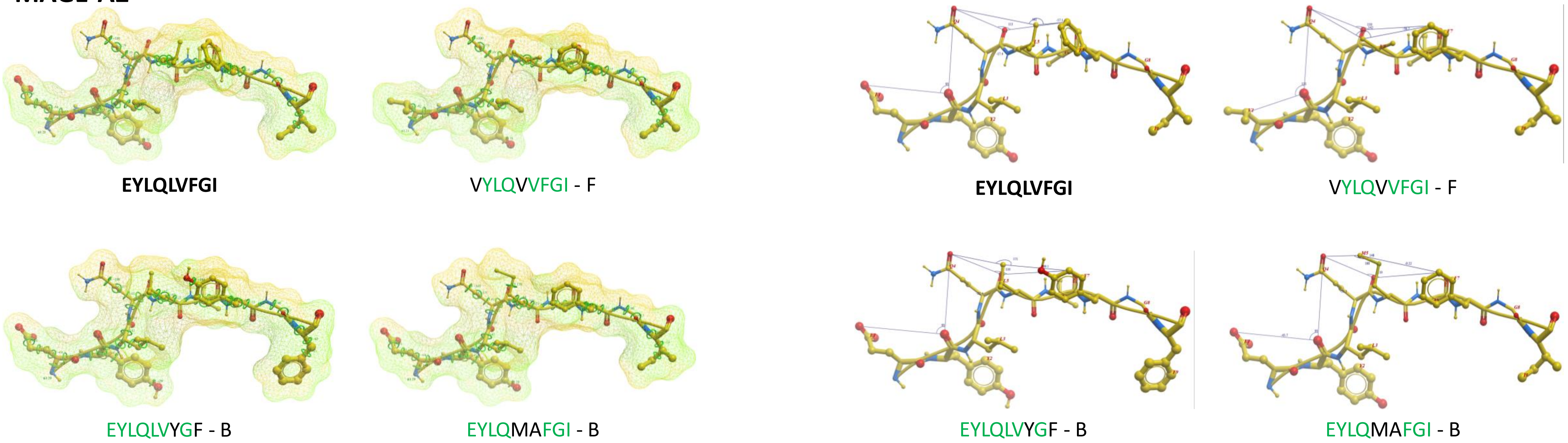

SAGE

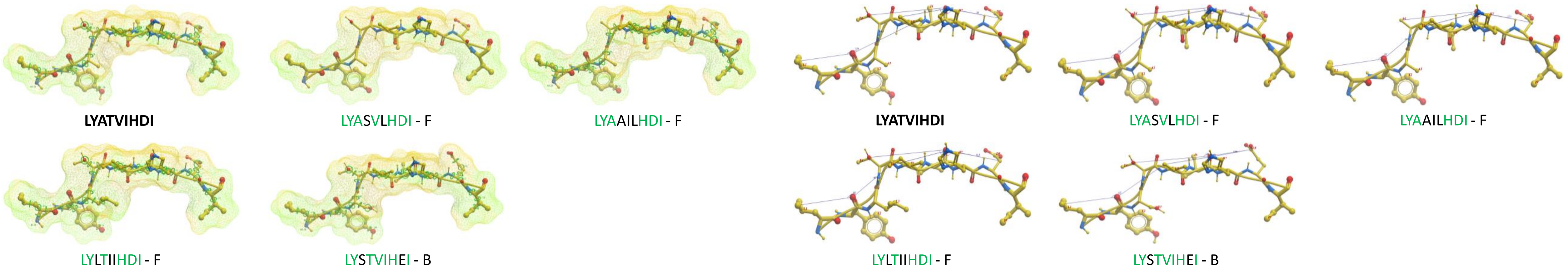

Supplement: Supplementary file 1 — Additional file 1: Figures S1–4. Percentage (average) of type of residues at each position of the microbiota-derived epitopes when aligned to the corresponding TAA. (A) Identical residues; (B) conservative residues; (C) non-conservative residues; (D) Identical + conservative residues. Figure S5. Affinity to HLA-A* 02:01 and 24:02 molecules of each microbiota-derived epitopes with homology to the corresponding TAA. The red dotted line indicates the affinity of the TAA. Figure S6–17. Predicted 3D conformation of TAA and microbiota-derived paired peptides. The surface conformation of the paired TAA microbiota-derived peptides is shown. Residues in the microbiota-derived epitopes identical to the TAA sequences are indicated in green color. Green areas = contact points with HLA-A molecule; Magenta areas = contact points with TCR α chain; Light Blue = contact points with TCR β chain. The latter are available only in Figure S6–S13. (HLA-A* 02:01 restricted epitopes). Figures S18. Percentage of identical values (± 10%) of planare and dehidral angles between of the microbiota-derived epitopes and the corresponding TAA. Figures S19–S24. Structural predicted backbone conformation of paired TAA and microbiota-derived epitopes. Green areas = contact points with HLA molecule; Magenta areas = contact points with the TCR α chain; light blue areas = contact points with the TCR β chain. The latter are available only in Additional file 1: Figure S19–22. (HLA-A* 02:01 restricted epitopes). Values of the planar as well as dihedral angles between atoms of selected TCR-facing residues are shown in parallel. [file 12967_2022_3512_MOESM1_ESM.pdf]
